# Inorganic Fillers in Composite Gel Polymer Electrolytes for High-Performance Lithium and Non-Lithium Polymer Batteries

**DOI:** 10.3390/nano11030614

**Published:** 2021-03-01

**Authors:** Vo Pham Hoang Huy, Seongjoon So, Jaehyun Hur

**Affiliations:** Department of Chemical and Biological Engineering, Gachon University, Seongnam 13120, Korea; vophamhoanghuy@yahoo.com.vn (V.P.H.H.); tjdwns7594@naver.com (S.S.)

**Keywords:** inorganic filler, gel polymer electrolytes, TiO_2_, Al_2_O_3_, SiO_2_, ZrO_2_, CeO_2_, BaTiO_3_, lithium polymer batteries

## Abstract

Among the various types of polymer electrolytes, gel polymer electrolytes have been considered as promising electrolytes for high-performance lithium and non-lithium batteries. The introduction of inorganic fillers into the polymer-salt system of gel polymer electrolytes has emerged as an effective strategy to achieve high ionic conductivity and excellent interfacial contact with the electrode. In this review, the detailed roles of inorganic fillers in composite gel polymer electrolytes are presented based on their physical and electrochemical properties in lithium and non-lithium polymer batteries. First, we summarize the historical developments of gel polymer electrolytes. Then, a list of detailed fillers applied in gel polymer electrolytes is presented. Possible mechanisms of conductivity enhancement by the addition of inorganic fillers are discussed for each inorganic filler. Subsequently, inorganic filler/polymer composite electrolytes studied for use in various battery systems, including Li-, Na-, Mg-, and Zn-ion batteries, are discussed. Finally, the future perspectives and requirements of the current composite gel polymer electrolyte technologies are highlighted.

## 1. Introduction

Electrolytes serve as the transportation medium for charge carriers between a pair of electrodes that are ubiquitous in electrolyte cells, fuel cells, and batteries [1]. For several decades, liquid electrolytes (LEs) have been employed extensively in electrochemical devices owing to their high electrolytic conductance (10^1^–10^2^ mS cm^−1^ for aqueous electrolytes and 10^0^–10^1^ mS cm^−1^ for organic electrolytes). However, the safety issues associated with LEs in terms of electrolyte leakage from flammable organic solvents have hindered the commercialization of lithium-metal (Li-metal) electrodes in lithium-ion batteries (LIBs) [2,3,4,5,6,7,8,9,10,11]. Another limitation associated with LEs is the inevitable dendrite growth of lithium due to uneven current when using porous separators [12,13,14,15,16,17,18]. Furthermore, increasing the energy density in LIBs using high-voltage materials often leads to electrode degradation as it requires electrode–electrolyte compatibility [19,20,21,22,23,24,25]. Therefore, the development of new electrolytes is essential to overcome the aforementioned issues in LIB applications.

**Figure 1:**
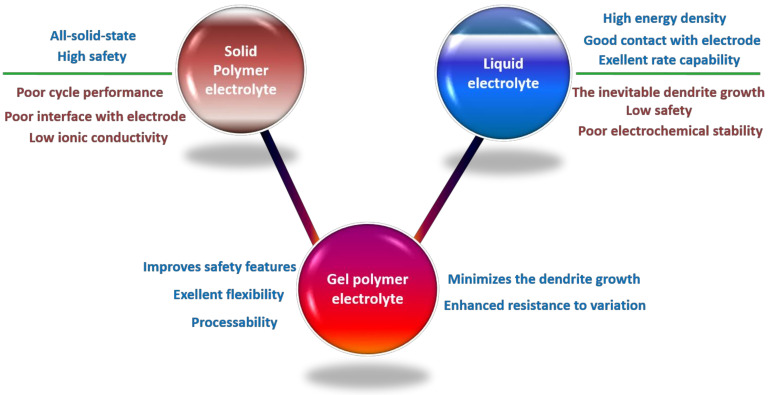
Advantages and disadvantages of solid polymer electrolytes (SPEs), liquid electrolytes (LEs), and gel polymer electrolytes (GPEs).

Solid polymer electrolytes (SPEs) without a liquid solvent have the potential to overcome the limitations associated with Les [26,27]. One of the interesting concepts recently demonstrated is “polymer-in-ceramic” configuration. Zhang et al. synthesized SPE with a flexible and self-standing membrane by incorporating 75 wt% ceramic particles into polymer matrix [28]. Poly(ε-caprolactone) (PCL) was proved to be a great polymer matrix that can accommodate high content of ceramic particles compared to other polymers due to its good mechanical strength and ability of structural rearrangement. The high concentration of ceramic in polymer-in-ceramic can be favorable in enhancing the ionic conductivity. For example, in poly(ethylene oxide)-garnet electrolyte SPE, Li^+^ ion transport is highly enhanced when the content of ceramic garnet reaches the percolation threshold. While the conductivity of the SPE is dominated by the polymer chain movement when the ceramic concentration is below the percolation threshold, the conductivity is boosted when ceramic concentration reaches the percolation threshold. This is because of the additional Li^+^ ion transport pathway formed by the ionic conducting ceramic. Therefore, above the percolation threshold, the presence of ceramic in SPE can dominantly affect the ionic conductivity [29]. However, the utilization of SPEs in electrochemical cells is still limited owing to their low ionic conductivities (10^−5^–10^−2^ mS cm^−1^), poor contact at the electrode-electrolyte interface, and narrow electrochemical window, resulting in a degenerated cyclic performance [30,31]. Recently, plastic crystalline electrolytes (PCEs) have been extensively studied for their high electrical conductivity, soft texture, and good thermal stability. Succinonitrile (SN)-based PCE is synthesized as an alternate layer to resolve the interfacial instability between solid electrolyte (SE) and Li metal, allowing for the widespread use of Li metal anode in the solid-state lithium batteries. Besides, chemical compatibility between SE and PCE ensures the prolonged life of solid-state batteries [32,33]. Tong et al. synthesized the SE interface by incorporating SN-based PCE in combination with salt and Li_7_La_3_Zr_1.4_Ta_0.6_O_12_ (LLZTO), which enhances surface stability for Li metal anode and Li_1.5_Al_0.5_Ge_1.5_(PO_4_)_3_ (LAGP)-based ceramic electrolyte [34]. The presence of the PCE interface helps to protect the ceramic electrolyte from reduction and promotes good interface stability with Li metal anode and LAGP due to its soft texture. In the absence of the PCE interface, the cell achieved a discharge capacity of 80 mAh g^−1^ at 0.05 C, whereas the cell with PCE interface showed an increase in discharge capacity of 126 mAh g^−1^ at 0.05 C. Thus, PCE that can act as a combination agent between the LAGP and Li metal anode prevents the penetration of dendrites into LAGP-based ceramic electrolyte. Compared with SPEs, gel polymer electrolytes (GPEs) are promising candidates for LIBs and non-LIBs as they combine the advantages of both LEs and SPEs in terms of ionic conductivity and mechanical properties [1,35,36,37,38,39,40,41]. Figure 1 shows the general advantages and disadvantages of LEs, SPEs, and GPEs.

**Figure 2:**
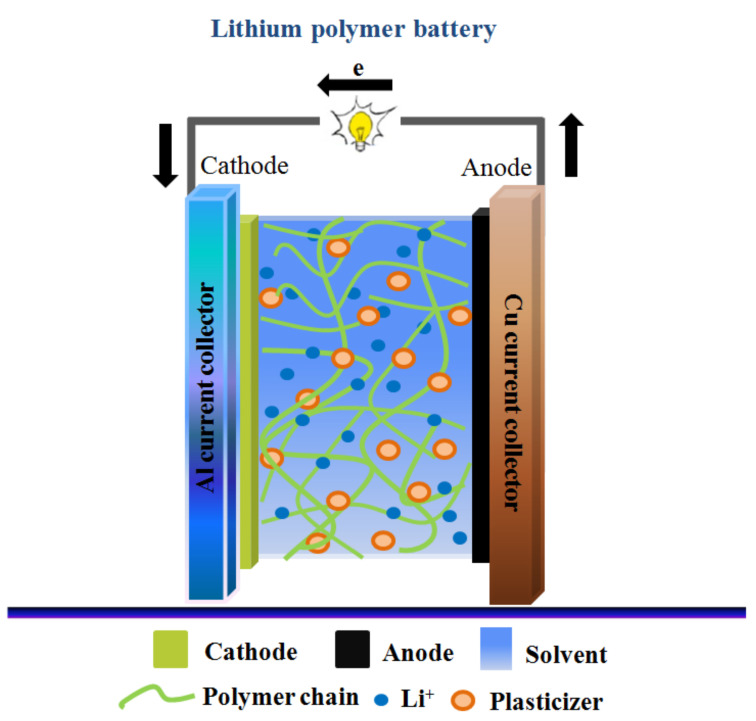
Schematic of a lithium polymer battery based on GPEs.

GPEs generally comprise a polymer matrix-lithium salt (Li-salt) system, small amount of integrated liquid plasticizer, and/or solvent, as shown in Figure 2 [42]. GPEs are characterized as homogeneous (uniform) and heterogeneous (phase-separated) gels. Typically, heterogeneous GPEs consist of a polymer framework of which the interconnected pores are filled with LEs. Thus, lithium ion (Li^+^ ion) transport mainly proceeds in the swollen gel phase or liquid phase in heterogeneous GPEs, which has a higher electrolytic conductance than SPEs. In addition, owing to their high safety and flexibility, GPEs are increasingly utilized for the manufacturing of advanced energy storage devices [43,44]. Several types of polymer matrices have been investigated as frameworks for GPEs, including polyethylene oxide (PEO), polyacrylonitrile (PAN), poly(vinylidene fluoride-co-hexafluoropropylene) (PVDF-HFP), poly(ethylene oxide-co-ethylene carbonate) (P(EO-EC)), poly(methyl methacrylate) (PMMA), poly(vinyl alcohol) (PVA), poly(vinyl chloride) (PVC), poly(propylene glycol) (PPG) [42,43,44,45]. In addition, the elastomeric polymer (polydimethylsiloxane (PDMS)) was used in the GPE nanocomposite due to its mechanical flexibility as well as chemical and thermal stability; thus, the nanocomposite PDMS-based membrane provides good electrochemical performance with high mechanical flexibility [46,47,48,49]. Owing to the combination of a polymer-salt system with a plasticizer, the mechanical strength of GPEs is mainly determined by the polymer matrix, while the plasticizer reduces the crystallized phase of the polymer matrix. This promotes segmental motion of the polymer matrix and affects the ionic conductivity of the GPE [50].

However, when incorporating an excess of plasticizer, it can deteriorate the mechanical strength of the film and its thermal stability, resulting in safety hazards [13,51]. Generally, blending, copolymerization, and crosslinking are used to improve the properties of polymer matrices and produce GPEs that perform well in LIBs. However, more importantly, the use of appropriate inorganic fillers in GPEs has recently emerged as one of the most promising methods to enhance the strength of the membranes, ionic conductivity, and Li^+^ ion transfer, which results in the GPEs performing well in LIBs [43].

Recently, a series of new approaches for incorporation of inorganic fillers have been developed to improve the electrochemical properties of GPEs. Besides, the incorporation of inorganic fillers in shape memory polymer (SMP) has been studied as an effective method for enhancing the mechanical properties and allowing multiple functionalities. SMP is a kind of material that can highly interact with stimulants such as temperature, light, and electromagnetic field to recover its original shape, suggesting this material as an important smart polymer which widely applied in industry [52]. In an attempt to enhance SMP’s recovery, Park et al. studied the effect of SiO_2_ fillers on polyurethane (PU) properties [53]. A cross-linking formed between the PU chains and hydroxide on the surface of SiO_2_ has improved the shape memory effect and enhanced the mechanical properties with 0.2 wt% SiO_2_. Besides, with the advantages of mechanical strengths as well as high elastic modulus, CNTs were used as effective fillers in improving the recovery of SMPs. CNTs-based nanocomposite showed excellent shape fixation ability with the recovery up to 90%, much higher than pristine SMP. Thus, SMP composite can be widely utilized in the industry [54,55]. GPEs have become one of the most effective electrolytes for applications such as wearable devices that require multiple functionalities, including flexibility, deformability, stretchability, and compressibility. Nevertheless, there has been a lack of discussion regarding the fundamental aspects of GPEs, including the materials used, preparation processes, their properties (mechanical, optical, and electrochemical), and their mechanism [56,57,58,59]. To obtain an overall comprehension of the recent studies on inorganic fillers and highlight their representative achievements, it is necessary to summarize the important findings for future studies on GPEs with an aim at developing high electrochemical energy storage properties.

In this review, the historical development of inorganic fillers in GPEs is first introduced. In the following sections, the details of inorganic fillers applied in GPEs along with various synthetic routes are presented. Subsequently, the mechanism of conductivity enhancement in the presence of inorganic fillers is discussed in detail. Finally, the application of GPEs in various battery systems (Li, Na, Mg, and Zn batteries) is discussed.

## 2. Historical Overview of GPEs

**Figure 3:**
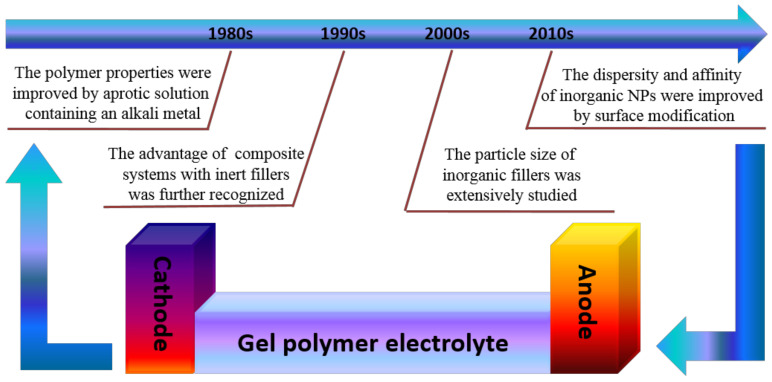
Historical overview of the developments of inorganic fillers in GPEs.

GPEs can be divided into three different categories based on the constituents of the mixture: plasticizer-added GPEs, inorganic filler-added GPEs, and a combination of plasticizer and inorganic filler-added GPEs. Lithium salt is responsible for the transportation of ions in the polymer framework, whereas the polymer accommodates the electrolyte to provide mechanical strength. The development of inorganic fillers in GPEs goes back to the early 1980s, when an attempt was made to improve the mechanical stability of the polymer matrix using an aprotic solution containing an alkali metal [60]. In the 1990s, the role of inert fillers in GPE composite systems started to be actively recognized. The addition of fillers to polymer segments has been reported to enhance Li^+^-polymer interactions and Li^+^ ion transport because of the dominant Li^+^ ion movement along the filler surface rather than through the polymer segment [61,62]. Since then, research on the incorporation of inorganic fillers into GPEs has rapidly expanded. In the 2000s, the effect of particle size of inorganic fillers in GPEs was extensively studied. Wang et al. prepared GPEs containing PVDF-HFP with a novel hierarchical mesoporous SiO_2_ network, which exhibited mechanical stability and higher ionic conductivity compared with GPEs without SiO_2_, or with fumed SiO_2_ [63]. Yang et al. synthesized SiO_2_ (m-SBA15) with enhanced ionic conductivity owing to the liquid electrolyte being trapped by the mesoporous structure and the large specific surface area of m-SBA15 [64]. In the 2010s, another effective method, surface modification, was proposed to improve the dispersion and affinity of inorganic fillers within organic compounds. A significant effort has been made to develop GPEs based on inorganic fillers, such as BaTiO_3_ [65], Al_2_O_3_ [66], TiO_2_ [67], ZnS [68], and CeO_2_ [69]. Figure 3 shows a historical overview of the development of inorganic fillers in GPEs.

The implementation of GPEs in a variety of battery systems, including LIBs and Li-sulfur batteries, has been increasingly studied. The presence of plasticizers in GPEs increases Li^+^ ion transfer; however, it simultaneously deteriorates the mechanical properties of the polymer matrix. Furthermore, electrode degradation may occur because of redox reactions between the plasticizer and electrodes. Consequently, to overcome the limitations of plasticizers, inorganic fillers have been proposed as additive materials to increase the electrolytic conductance and mechanical stability of GPEs. Osinska et al. [70] modified the surface of inorganic fillers to improve electrolyte absorption, thereby providing a more favorable medium for ion transport. A new ion transport pathway was discovered by Kumar et al. [71] by introducing spatially charged layers that tend to overlap with the concentrated filler grains dispersed in GPEs. The polymer chain containing active sites and surface groups of the inorganic fillers are affected by Lewis acid–base interactions. This interaction mostly occurs between the carbonyl groups of poly(acrylamide) (PAM) and surface groups of Al_2_O_3_, resulting in changes in the morphology of the GPEs [72].

## 3. Details of the Inorganic Fillers Applied in GPEs

The key functionalities of inorganic fillers in GPEs are to enhance Li^+^ ion transfer and mechanical stability, wherein the polymer provides the conduction pathway for the ions, whereas the fillers influence the physical durability of the polymer to support ion transport. In addition, the inorganic filler particles can be used as a “solid plasticizer,” which reduces the crystallinity of the host polymer and increases the transport properties. In this section, the addition of different types of inorganic fillers to enhance the mechanical properties and electrolytic conductance of GPEs is described in detail. Various inorganic fillers are listed according to research prevalence from the early 2000s to the present.

### 3.1. Titanium Dioxide (TiO_2_)

**Figure 4:**
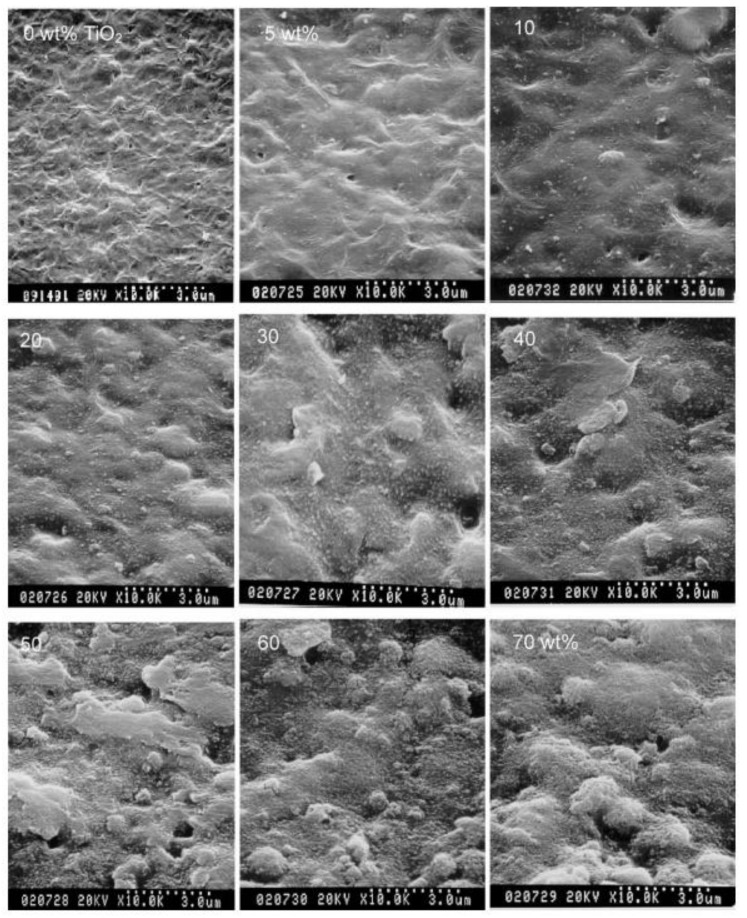
Surface morphology of P poly(vinylidene fluoride-co-hexafluoropropylene) (PVDF-HFP) with varying TiO_2_ (rutile) contents from the upper left to the lower right panel. Reprinted with permission from Kim et al. [74]. Copyright 2003 Elsevier B.V.

Chung et al. [73] investigated the effect of TiO_2_ nanoparticle (NP) addition in (PEO)-LiClO_4_ for the improvement of electrochemical performance of GPEs. This study provided a model for the effects of inorganic fillers on the overall Li^+^ ion transport in nanocomposite electrolytes. In addition, the specific role of inorganic filler was interpreted in terms of Lewis acid-base interactions. Over a wide temperature range, two structural modifications occurred at the ceramic surface. First, the morphology of the polymer was modified by the structural groups on the surface of the particles, which provided crosslinking opportunities for the PEO segments and X-anion. This resulted in a reduction in the energy barrier of the reorganized PEOs, where appropriate Li^+^ ion-conducting pathways were established at the ceramic surface. Second, an “ion-ceramic complex” was formed through the dissociation of salt due to the interaction between polar groups on the surface of the fillers and ions of the electrolyte. These two structural variations can account for the improvement in the electrolytic conductance of inorganic fillers in GPEs. Liu et al. [67] reported that GPEs with a TiO_2_ ceramic filler exhibited higher Li^+^ ion transfer numbers than GPEs without TiO_2_. The interaction between the fillers, anions, and polymer chains enhanced Li^+^ ion transfer. Kim et al. [74] investigated the influence of filler content on the morphology of GPE membranes. As the TiO_2_ content increased, the surface of the membrane coarsened, and small aggregates appeared; however, the TiO_2_ NPs remained well distributed over the entire surface area of the membrane with an increase in the content to 60 wt% (Figure 4). In addition, the ionic conductivity was enhanced owing to the nanopores in the liquid medium, as well as the effective ion transport achieved by the presence of TiO_2_. Therefore, the addition of rutile TiO_2_ NPs not only enhanced the dispersion of the constituents but also improved the physical and electrochemical properties of the GPE. Karlsson et al. [75] studied polymer kinetics using quasi-elastic neutron scattering experiments to observe the effect of the filler on the crystallinity and structural changes in the polymer. The results showed that the improvement in ionic conductivity was because of the filler rather than the enhanced polymer dynamics based on the presence of a 5-nm-thick immobilized polymer layer around the filler particles. Kwak et al. [76] prepared a viscous P(EO-EC)/LiCF_3_SO_3_/TiO_2_ polymer electrolyte mixture with a porous P(VdF-HFP)/P(EO-EC)/TiO_2_ membrane. The TiO_2_ content used was 0.0, 0.5, 1.0, 1.5, 2.0, 5.0, and 10.0 wt% in the polymer electrolyte and 0.0, 10.0, 20.0, 30.0, and 40.0 wt% in the porous membrane with a blend composition of 6:4 P(VdF-HFP) to P(EO-EC). The stress and tensile modulus values showed an increase for the membrane up to 2.0 and 41.0 MPa, respectively, using 30 wt% TiO_2_, and a decrease up to 1.2 and 34.5 MPa, respectively, when adding 40 wt% TiO_2_. These results indicated that the presence of high concentrations of TiO_2_ up to 20 wt% improved the mechanical properties of the membrane owing to the interaction between the NPs and the host polymer. In addition, an increase in ionic conductivity was observed up to 4.7 × 10^−2^ mS cm^−1^ at 25 °C for the GPEs containing 1.5 wt% TiO_2_ owing to the interaction between the oxide groups of the polymer and hydroxyl groups of the TiO_2_ filler.

**Figure 5:**
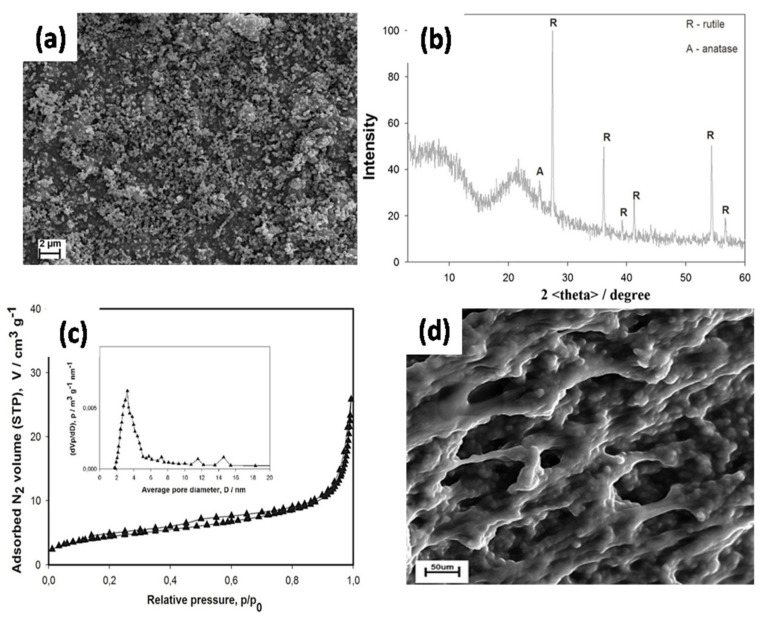
(**a**) SEM image of the TiO_2_-SiO_2_ hybrid composite, (**b**) wide angle X-ray spectroscopy of the TiO_2_-SiO_2_ hybrid composite, (**c**) nitrogen adsorption/desorption isotherm and pore size of the TiO_2_-SiO_2_ hybrid composite, and (**d**) SEM image of the surface of the membrane containing the TiO_2_-SiO_2_ hybrid composite. Reprinted with permission from Kurc et al. [81]. Copyright 2014 Elsevier Ltd.

Hwang et al. [77] reported that the electrolytic conductance of GPEs was increased when reducing the particle size of the TiO_2_. The ionic conductivity of a GPE containing TiO_2_ NPs was higher than that of a GPE without TiO_2_ at 30 °C. Agnihotry et al. [78] examined the effects of different concentrations of nanosized TiO_2_ in a PMMA-based GPE. This study demonstrated that the ionic conductivity was enhanced when using an optimum TiO_2_ loading (2 wt%) in the GPE. Byung et al. [79] synthesized a GPE by blending poly(acrylonitrile)-poly(ethylene glycol diacrylate) (PAN-PEGDA) with Li-salt and TiO_2_ NPs. The high surface area of inorganic fillers can improve the interfacial resistance and ionic conductivity of Li metal owing to an increase in chemical stability and possible retention of organic solvents in the micropores; this assists in the regulation of possible side reactions associated with Li metal. In addition, nanosized inorganic fillers were evenly dispersed and increased the mechanical stability of the polymer matrix. Walkowial et al. [80] and Kurc et al. [81,82] modified the surface of a new hybrid TiO_2_/SiO_2_ filler for GPEs. The original hybrid fillers were modified by grafting functional groups, such as methacryloxy or vinyl groups, on the surface of the fillers. The surface modification chemistry of the filler seemed to be another factor affecting the overall performance of the GPE in terms of solvent absorption, specific conductivity, and intercalation of lithium on graphite. As shown in the SEM images (Figure 5a), the hybrid TiO_2_/SiO_2_ spherical NPs are homogeneously dispersed. Figure 5b shows that the hybrid TiO_2_-SiO_2_ predominantly contains rutile TiO_2_. Figure 5c indicates that the mean pore diameter of the TiO_2_-SiO_2_ hybrid is 3.8 nm, which represents the mesoporous components, and the surface area is 12.5 m^2^/g, which suggests an intermediate surface activity level. Hybrid TiO_2_/SiO_2_ powder with a moderate degree of surface functionalization was considered as a potential candidate for GPE applications in LIBs. The SEM image of the GPE without functionalized fillers typically exhibits a porous structure (Figure 5d).

An experimental investigation by Yahya et al. was performed on proton-conducting GPE nanocomposites based on TiO_2_ NP-dispersed cellulose acetate (CA) [83]. The increase in the ionic conductivity of the GPE nanocomposite could be explained by the addition of TiO_2_, which increased the total solution/solvent dielectric constant. The dielectric constant of TiO_2_ was higher than that of N,N-dimethylformamide (DMF), leading to a reduced Coulombic interaction between the ion aggregates and dissociation of ions from the salt resulting in free NH_4_^+^ ions. The GPE prepared by adding NH_4_BF_4_ and TiO_2_ to CA was considered a promising material for proton batteries.

**Figure 6:**
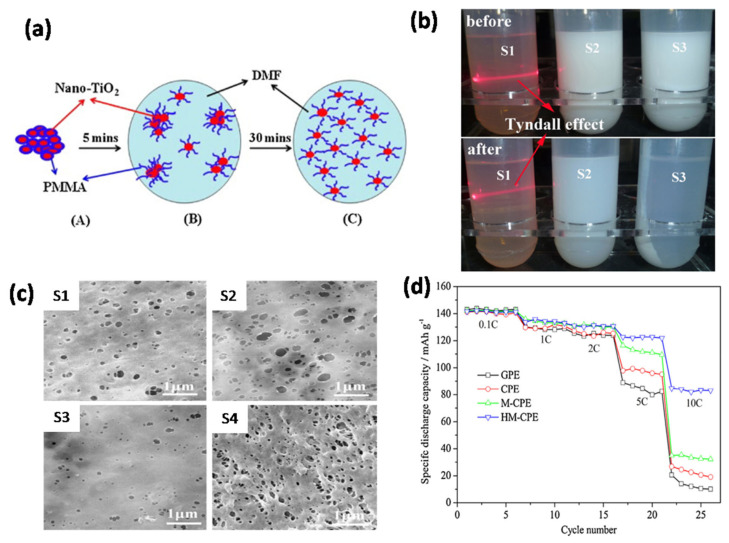
(**a**) Schematic of nano-TiO_2_- poly(methyl methacrylate) (PMMA) in N,N-dimethylformamide (DMF) (A: mixture of nano-TiO_2_ and PMMA, B: tethered PMMA on Nano-TiO_2_, C: self-assembly of PMMA-tethered Nano-TiO_2_) and (**b**) photographs of the dispersed NPs in DMF before (top panel) and after (bottom panel) 10 min centrifugation at 10,000 rpm. The same TiO_2_ content (5 wt%) was used in each sample: (S1) highly dispersed nano-TiO_2_-PMMA, (S2) nano-TiO_2_-PMMA, and (S3) pristine nano-TiO_2_. (**c**) SEM images of the GPEs: (S1) pristine PVDF-HFP (“GPE”), (S2) nano-TiO_2_/PVDF-HFP (“CPE”), (S3) nano-TiO_2_-PMMA/PVDF-HFP (“M-CPE”), and (S4) highly dispersed nano-TiO_2_-PMMA/PVDF-HFP (“HM-CPE”). (**d**) The rate capabilities of the different electrolytes shown in (**c**). Reprinted with permission from Chen et al. [85]. Copyright 2013 Elsevier Ltd.

**Figure 7:**
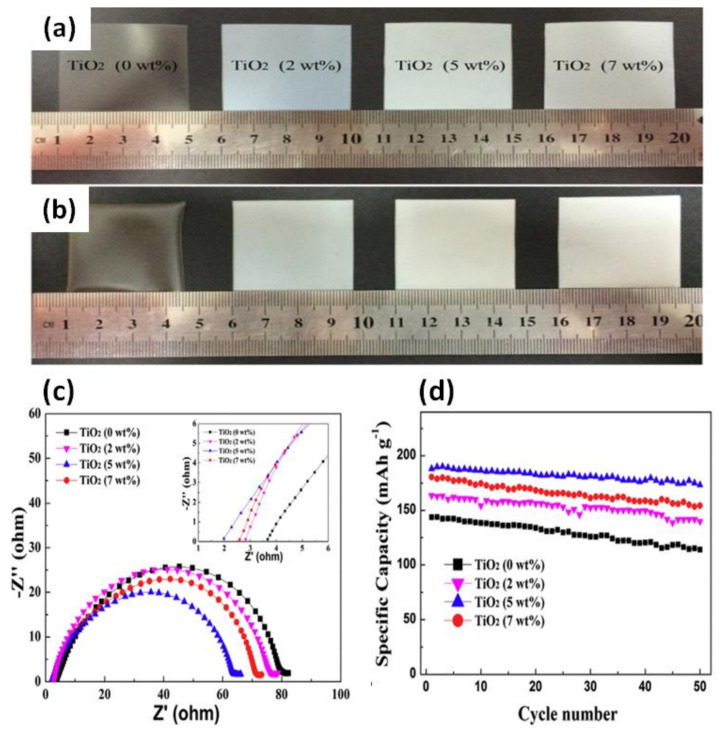
Thermal behavior of the GPE membranes (**a**) before and (**b**) after storing at 130 °C for 1 h, (**c**) EIS results of the GPE membrane containing varying contents of TiO_2_, and (**d**) cyclic performance of the LiCoO_2_/Li cells using the GPE at 0.2 C. Reprinted with permission from Chen et al. [88]. Copyright 2015 Elsevier B.V.

Cao et al. [84] demonstrated improvement in the GPE by incorporating TiO_2_ in PVDF/PMMA via electrospinning for practical applications in LIBs. The GPE containing 3 wt% TiO_2_ showed a highest electrolytic conductance of 3.9 × 10^−1^ mS cm^−1^ with an electrochemical stability up to 5.1 V vs. Li^+^/Li at room temperature. The increase in electrolytic conductance with the addition of TiO_2_ particles was due to better dispersion through a Lewis acid–base interaction between the polar groups of the electrolytes and the filler and a decrease in the crystallinity of the polymer. Hong Chen et al. [85] investigated the role of nano-TiO_2_ dispersibility in a GPE based on a PVDF-HFP polymer for LIBs (schematic is shown in Figure 6a). Figure 6b shows the dispersion of the nanoparticles (pristine, commercial, and modified TiO_2_) in DMF. The morphology of nano-TiO_2_-PMMA doped PVDF-HFP membrane exhibits a smoother surface with fewer pores compared to the pristine PVDF-HFP membrane and nano-TiO_2_ doped PVDF-HFP membrane. Interestingly, the highly dispersed TiO_2_-PMMA hybrid membrane has a rougher surface, and the pore size is more uniform (Figure 6c). The effect of TiO_2_ dispersion on the C-rate discharge performance is shown in Figure 6d. The addition of nanosized TiO_2_ to the PVDF-HFP-based GPE significantly improved the discharge capacity of the cells. In addition, the highly dispersed nano-TiO_2_-PMMA doped GPE showed the highest discharge capacity compared to the other electrolytes. This study demonstrated that the dispersion of nanosized TiO_2_ is an important factor that influences the performance of the PVDF-HFP-based polymer electrolyte. Sankaranarayanan et al. [86] explained the effect of TiO_2_ on the electrochemical performance based on the distribution and aggregation of the fillers, Lewis acid–base interactions, and polymer segment-ion coupling. The addition of TiO_2_ to the GPE nanocomposite promoted Lewis acid–base interactions, thereby facilitating the dissolution of LiClO_4_ salts and increasing the amount of free Li^+^ ions. Bozkurt et al. [87] produced polymer electrolyte nanocomposites based on borate ester graft copolymer PVA, poly(ethylene glycol) methyl ether (PEGME)), nano-TiO_2_, and trifluoromethane sulfonate (CF_3_SO_3_Li). The conduction pathway for ion transport was improved by the boron-containing PVA backbone and flexible side chains. This study suggested that the presence of inorganic fillers resulted in an increase in ionic conductivity, Li^+^ ion transfer, interfacial stability between the electrode and electrolyte, and the mechanical strength of the GPEs. Chen et al. [88] showed that after adding TiO_2_ NPs, the PVDF-HFP/PMMA/TiO_2_ membrane exhibited enhanced thermal stability and electrolytic conductance. Based on the images of the GPE membrane, its thermal stability was significantly improved after the addition of TiO_2_ NPs (Figure 7a,b). In particular, the GPE containing 5 wt% TiO_2_ NPs showed homogeneously interconnected pores which resulted in an excellent performance (Figure 7c,d). The EIS analysis of GPE containing varying concentrations of TiO_2_, shown in Figure 7c, shows that after the introduction of TiO_2_, the bulk resistance (R_b_) of the GPEs significantly decreases for the sample containing the lowest TiO_2_ content of 5 wt%. The initial discharge capacities were 143.6, 180.5, 188.1, and 163.6 mAh g^−1^ for the different TiO_2_ contents (0, 2, 5, and 7 wt%), respectively. After 50 cycles, the capacity decreased to 114, 154.3, 173.2, and 139.9 mAh g^−1^, with a capacity retention of 79.4%, 85.5%, 92.1%, and 85.5%, respectively (Figure 7d). This study showed that the incorporation of TiO_2_ NPs to the GPE improved its electrochemical stability and ionic conductivity in LIBs. Yamolenko et al. [89] extended this study on the effect of NPs to a polyester-diacrylate (PEDAC)-based network polymer electrolyte. The mechanical strength of the GPE was enhanced after improving the distribution of the NPs in the polymer electrolyte by ultrasonic treatment compared to simple mechanical stirring.

**Figure 8:**
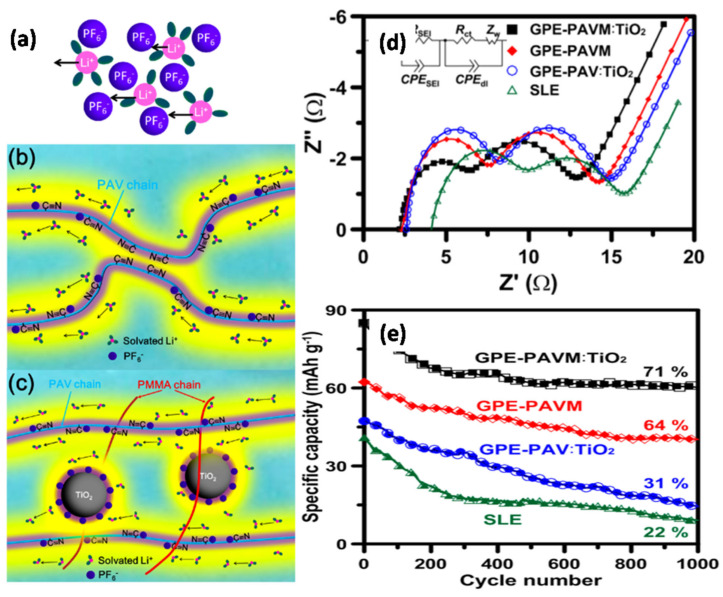
(**a**) Separator-supported LE (SLE): limitation in ionic dissociation. (**b**) GPE- poly(acrylonitrile-co-vinyl acetate) (PAV): a space-charge layer of Li^+^ ions is formed due to the PF_6_^−^ anion absorbed by the nitrile functional groups on the PAV chain. (**c**) GPE-PAVM:TiO_2_: the 3D percolation pathway is formed by the space-charged layers surrounding the TiO_2_ NPs and PAV chain. (**d**) Nyquist plots of the full cell (graphite-GPE-LFP) and (**e**) discharge capacity of the full cell at a 20 C-rate over a voltage range of 2.0–3.8 V. Reprinted with permission from Teng et al. [90]. Copyright 2016 American Chemical Society.

Teng et al. [90] reported a GPE prepared by combining poly(acrylonitrile-co-vinyl acetate) (PAV) with PMMA and TiO_2_ NPs, i.e., PAVM:TiO_2_ (Figure 8). This study introduced a new concept concerning the association of oxide NPs with the space-charge regimes around the polymer’s functional groups to induce 3D conduction pathways for Li^+^ ions in GPEs applied in LIBs (Figure 8a–c). Based on the impedance spectra shown in Figure 8d, the high-frequency semicircle represents the movement of charge carriers through the SEI layer, middle-frequency semicircle shows charge-transfer resistance, and sloping line is related to the Warburg impedance. The results revealed the superiority of GPE-PAVM:TiO_2_ in enhancing Li^+^ ion transport. Figure 8e presents the discharge capacities of the full-cell batteries at a high rate of 20 C. The cell with the GPE-PAVM:TiO_2_ electrolyte delivered discharge capacities of 152 and 84 mAh g^−1^ at 0.1 and 20 C, respectively, thus outperforming the cell with the SLE electrolyte with capacities of 146 and 40 mAh g^−1^, respectively. The Li^+^ ion transport efficiency in the bulk solution as well as at the electrode–electrolyte interface was enhanced through the 3D percolation pathway by immobilizing the PF_6_^−^ anions in the oxide NP framework. Sakunthala et al. performed a comparative study between single-crystalline TiO_2_ nanorods and submicronsized TiO_2_ fillers in PVDF-HFP/EC/LiClO_4_. The Li^+^ ion transfer and tensile strength of the membrane containing 5 wt% TiO_2_ nanorods were higher than those of the membrane containing 5 wt% submicronsized TiO_2_. This can be explained by the improved interaction of the rod-shaped morphology of the single-crystalline TiO_2_ filler with the polymer/salt/plasticizer matrix in the GPE [91].

Sivakumar et al. [92] fabricated a GPE containing a PVC-PEMA blend using hydrothermally derived TiO_2_ NPs as an inorganic filler. The influence of the filler NPs on the surface morphology, thermal stability, and electrochemical properties were studied. The addition of TiO_2_ NPs reduced the crystallinity of the polymer and enhanced the Li^+^ ion transport pathways. Similarly, Singh et al. [93] modified the structural properties of PEMA-based plasticized polymer electrolytes by incorporating TiO_2_ NPs. The addition of TiO_2_ NPs in the plasticized polymer electrolyte suppressed the crystallinity and enhanced the amorphous nature. Hence, the addition of TiO_2_ NPs could be a novel approach to enhance the electrolytic conductance in GPEs. The ionic conductivity and temperatures of some significant GPEs containing TiO_2_ fillers are listed in Table 1.

### 3.2. Aluminum Oxide (Al_2_O_3_)

**Figure 9:**
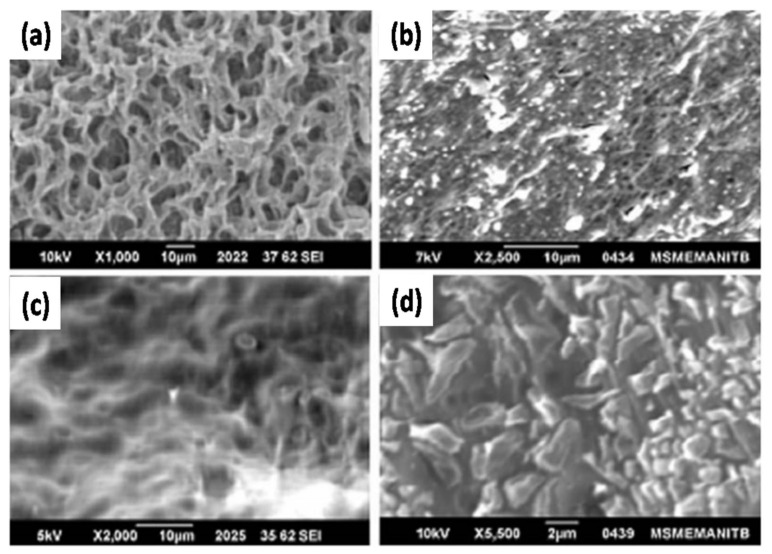
SEM images of the (**a**) PVA:NH_4_SCN/DMSO GPE containing (**b**) 2, (**c**) 6, and (**d**) 10 wt% Al_2_O_3_ NPs. Reprinted with permission from Rat et al. [97]. Copyright 2012 Indian Academy of Sciences.

Li et al. [94] prepared a GPE by combining porous P(VDF-co-HFP) with alumina (Al_2_O_3_) NPs as the filler. An increase in the Al_2_O_3_ NP content reduced the level of crystallization in the polymer, thereby increasing the amorphous phase of the membrane. Piotrowska et al. [95] described the effect of oxide fillers on the properties of GPEs with a PVDF-HFP polymer matrix. Modification of the PVDF-HFP membranes with Al_2_O_3_ NPs led to a decrease in the liquid phase uptake ability owing to the reduction of accessible pore spaces. Egshira et al. [96] assessed the availability of the alumina filler in an imidazolium-based gel electrolyte. The Al_2_O_3_ filler enhanced Li^+^ ion mobility by providing alternative pathways for Li^+^ ion movement and changing the interaction between the Li^+^ ions and the EO chain. Rai et al. [97] prepared nano-Al_2_O_3_-filled PVA composite gel electrolytes. As shown in Figure 9a, the PVA membrane exhibits a porous structure, while the addition of 2 wt% Al_2_O_3_ NPs reduces the porosity of the PVA composite electrolyte as the Al_2_O_3_ NPs are trapped among the chains in the pores (Figure 9b). Upon the addition of 6 wt% Al_2_O_3_ NPs (Figure 9c), the PVA chains are fully covered with Al_2_O_3_ NPs. This indicates complete dispersion of the Al_2_O_3_ nanofiller in the electrolyte film. Upon further addition of Al_2_O_3_ NPs (10 wt%), the grain sizes and shapes become irregular resulting in a partially crystalline structure containing Al_2_O_3_ NPs and PVA electrolyte (Figure 9d). An increase in the Al_2_O_3_ NP content increased the amorphous phase of pristine PVA. The Li^+^ ion transfer capacity of the GPE reached its maximum with the addition of 6 wt% Al_2_O_3_ NPs.

**Figure 10:**
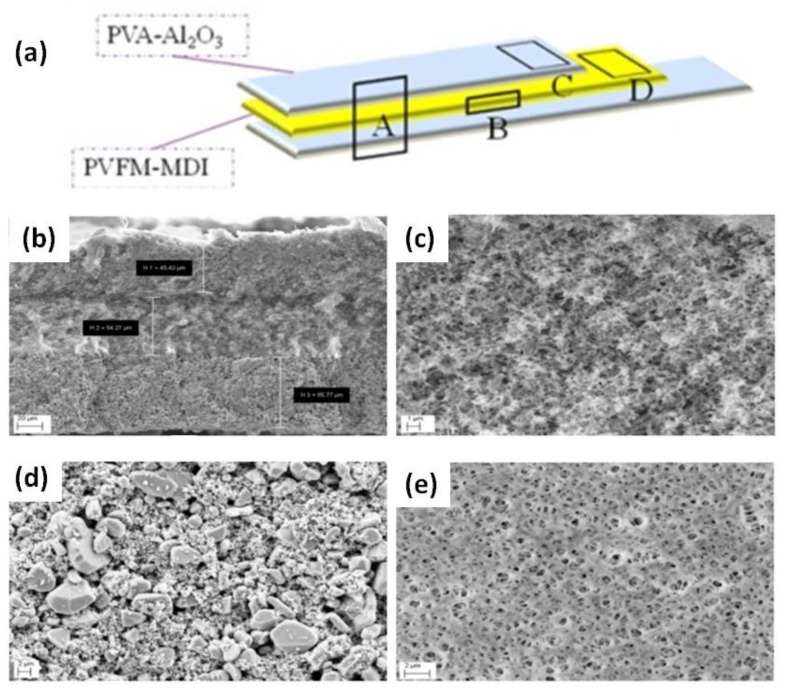
(**a**) Schematic of the membrane (Al_2_O_3_/PVFM/Al_2_O_3_). SEM images of the trilayer membrane; (**b**) cross-sectional image of the trilayer membrane; (**c**) cross-sectional image of the PVFM membrane; (**d**) the surface morphology of the Al_2_O_3_ coating layer; and (**e**) the surface morphology of the PVFM-based membrane. Reprinted with permission from Wen et al. [99]. Copyright 2007 Scientific Research Publishing Inc.

A novel GPE was prepared by combining a poly(methyl methacrylate-acrylonitrile-ethyl acrylate) (P(MMA-AN-EA)-based polymer electrolyte with nano-SiO_2_ and Al_2_O_3_ as inorganic fillers [98]. The maximum electrolytic conductance of the GPE was achieved when 5 wt% nano-SiO_2_ and nano-Al_2_O_3_ were used. This study showed the different roles of the inorganic fillers: SiO_2_ contributed to the enhanced ion conduction due to strong Lewis acid–base interactions, whereas Al_2_O_3_ improved the structural and thermal stability of the GPE owing to its high stiffness. Wen et al. [99] synthesized a novel GPE with a trilayer structure consisting of polyvinyl formal (PVFM)-4,4-diphenyl-methane diisocyanate (MDI) covered by a PVA-Al_2_O_3_ solution on both sides to achieve synergistic effects for each layer (Figure 10a). The morphology of the Al_2_O_3_/PVFM/Al_2_O_3_ trilayer membrane was determined by FESEM (Figure 10b–e). The thicknesses of the layers were 45.42, 54.27, and 65.77 µm, respectively (Figure 10b). The morphology of the PVFM membrane exhibits sponge-like pores, which are expected to enhance the transport of Li^+^ ions (Figure 10c). The porous structure of the inorganic particulate films did in fact increase Li^+^ ion transfer (Figure 10d). The surface morphology of the PVFM membrane shows evenly distributed pores, as shown in Figure 10e. This study indicated that the inorganic layers enhanced the thermal integrity and mechanical properties of the membrane. This multilayer polymer membrane could be a potential material for application in LIBs.

**Figure 11:**
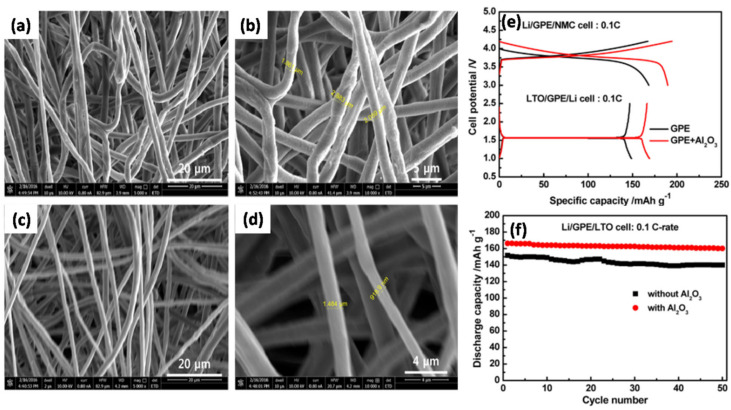
Morphology of (**a**,**b**) pristine GPE and (**c**,**d**) Al_2_O_3_-GPE membrane. (**e**) Initial charge–discharge curves of the Li_4_Ti_5_O_12_ (LTO) and LiNi_1/3_Mn_1/3_Co_1/3_O_2_ (NMC) half cells and (**f**) the cyclic performance of the GPE membrane. Reprinted with permission from Kim et al. [100]. Copyright 2017 Elsevier Ltd.

Kim et al. [100] prepared a GPE containing a PVDF-HFP fibrous matrix with Al_2_O_3_ as the inorganic filler by electrospinning. The morphologies of polymer fibrous matrices exhibiting different diameters are shown in Figure 11a–d. The surface morphology of the pure polymer is rougher than that of the Al_2_O_3_-composite polymers and exhibits an average diameter of 2.3 µm (Figure 11a,b) compared to 1.2 µm for the Al_2_O_3_-composite polymer matrix (Figure 11c,d). The presence of inorganic fillers in the fibrous polymer matrices prevented polymer shrinkage and agglomeration, which was beneficial for the formation of homogeneous pores. Figure 11e shows that the discharge capacities of the NMC and LTO half cells of the GPE-Al_2_O_3_ composite are 189.6 and 166.3 mAh g^−1^, respectively, which are higher than those of the GPE without Al_2_O_3_ (168.2 and 146.8 mAh g^−1^, respectively). The presence of Al_2_O_3_ NPs increased the porosity and absorption of free ions, thereby enhancing the electrolytic conductance and electrochemical stability compared to a pure GPE. The Al_2_O_3_-composite GPE also showed a better discharge capacity retention of ~96% (initial and final specific capacities of 166.3 and 160.2 mAh g^−1^, respectively) (Figure 11f). Jain et al. [101] presented efficient conduction pathways constructed in PVdF-based GPEs with Al_2_O_3_ and boron nitride (BN) ceramic nano/microparticles. The high dielectric constants of Al_2_O_3_ and BN facilitated anion capture in the GPE and the transfer of Li^+^ without coordination to the anions.

**Figure 12:**
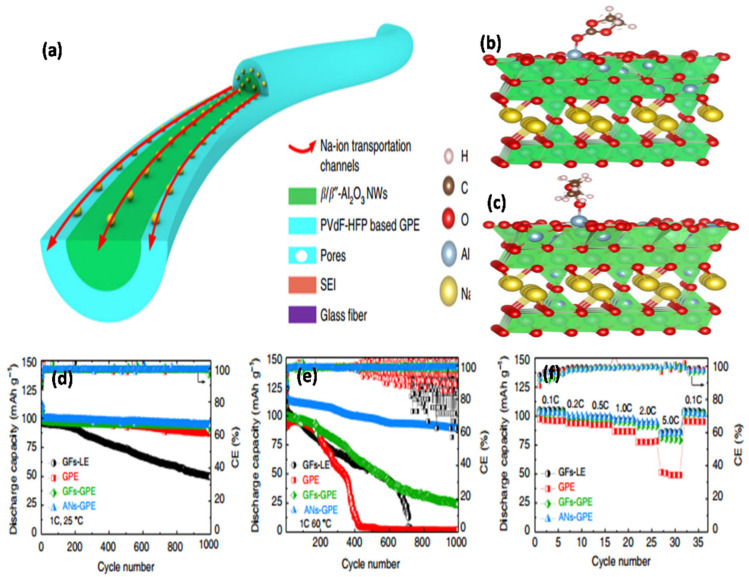
(**a**) Schematic of Na-ion transportation in the Al_2_O_3_ nanowire (AN)-GPE. (**b**,**c**) Adsorption of ethylene carbonate (EC) and diethylene carbonate (DEC) on the β-Al_2_O_3_ (003). (**d**,**e**) Cyclic performance of the Na_3_V_2_(PO_4_)_3_ (NVP)/Na cells using a glass fiber (GF)-LE, GPE, GFs-GPE, and ANs-GPE at 1 C under 25 and 60 °C, respectively. (**f**) Rate performance of the NVP/Na cells using the different GPEs. Reprinted with permission from Yang et al. [104]. Copyright 2019 Springer Nature.

Delgado-Rosero et al. [102] synthesized a GPE containing PEO and sodium trifluoroacetate (CF_3_COONa) with different contents of Al_2_O_3_. The addition of inorganic fillers increased the amorphous phase portion surrounding the filler of the (PEO)_10_CF_3_COONa + x wt% Al_2_O_3_ composite, thereby improving Na^+^ ion transport through the pathways of the amorphous phase. Maragani et al. [103] reported a GPE containing a combination of PAN and sodium fluoride (NaF) with Al_2_O_3_ nanofibers formed through a solution casting technique. With an increase in the Al_2_O_3_ nanofiber content, the amorphous phase of the GPE increased, resulting in an improvement in ion conduction. Yang et al. [104] designed a novel GPE with uniformly cross-linked β-Al_2_O_3_ nanowires that compactly covered a P(VDF-co-HFP)-GPE through strong molecular interactions (Figure 12a–c). In this innovative structure, the LEs were immobilized through bonding between the cross-linked Al_2_O_3_ nanowires and PVDF-HFP (ANs-GPE), thereby creating uniform and continuous Na^+^ ion transport channels along the Al_2_O_3_ nanowires (Figure 12a–c). This innovative structure can significantly improve the density and homogeneity of the Na^+^ ion transport channel, resulting in superior electrochemical performance (Figure 12d–f). Mishra et al. [105] studied the effect of Al_2_O_3_ NP dispersion on a PVdF-HFP/PMMA blend-based nanocomposite GPE system. The electrolytic conductance changed significantly depending on the Al_2_O_3_ concentration in the PVdF-HFP/PMMA membrane. The maximum electrolytic conductance achieved was ~1.5 × 10^0^ mS cm^−1^ when 6 wt% Al_2_O_3_ NPs were added to the GPE. The ionic conductivities and temperatures of the GPEs containing Al_2_O_3_ fillers are summarized in Table 2.

### 3.3. Silicon Dioxide (SiO_2_)

**Figure 13:**
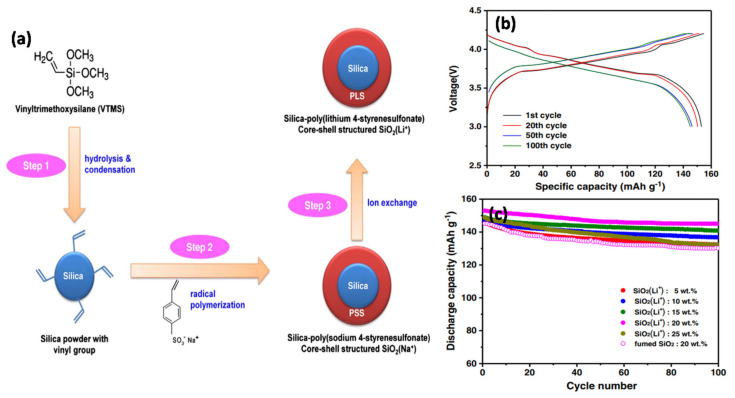
(**a**) Reaction scheme for the synthesis of the SiO_2_(Li^+^) particles. (**b**) Charge-discharge curves of the GPE containing 20 wt.% SiO_2_(Li^+^) particles. (**c**) Discharge capacities of the GPE containing different contents of SiO_2_(Li^+^) particles. Reprinted with permission from Kim et al. [108]. Copyright 2012 Elsevier B.V.

Wieczorek et al. [106] designed a novel model GPE using a combination of amorphous poly(ethylene oxide) dimethyl ether (PEODME) and LiClO_4_ with fumed nano-silica. The presence of nanosized fumed silica in the GPE was promising because of the reduction in ion association. Wu et al. [107] prepared a hybrid polymer electrolyte film consisting of PMMA, LiClO_4_, propylene carbonate (PC), and SiO_2_ filler using a solvent casting technique. The conductivity was not positively correlated with the increased concentration of SiO_2_ owing to the aggregation of SiO_2_, which led to the formation of crystal-like particles on the surface of the membrane. Kim et al. [108] reported novel homogeneous spherical core-shell structured SiO_2_(Li^+^) NP fillers that were applied as functional fillers in GPEs (Figure 13). SiO_2_(Li^+^) was synthesized by dispersing Li^+^ ions in the core-shell structure of the SiO_2_ particles (Figure 13a). Figure 13b shows the charge–discharge curves of the GPE containing 20 wt% SiO_2_(Li^+^). The first discharge capacity with LiCoO_2_ in the cathode was 153 mAh g^−1^. The GPEs containing the novel filler exhibited unique Li^+^ ion transport and mechanical strength. Consequently, the battery exhibited low internal resistance, high capacity, and a stable cyclic performance. In addition, the capacity retention was enhanced by increasing the SiO_2_(Li^+^) content in the GPEs up to 20 wt% (Figure 13c). The addition of SiO_2_(Li^+^) particles resulted in the retention of more LEs in the GPE, thereby improving the electrochemical performance during cycling.

**Figure 14:**
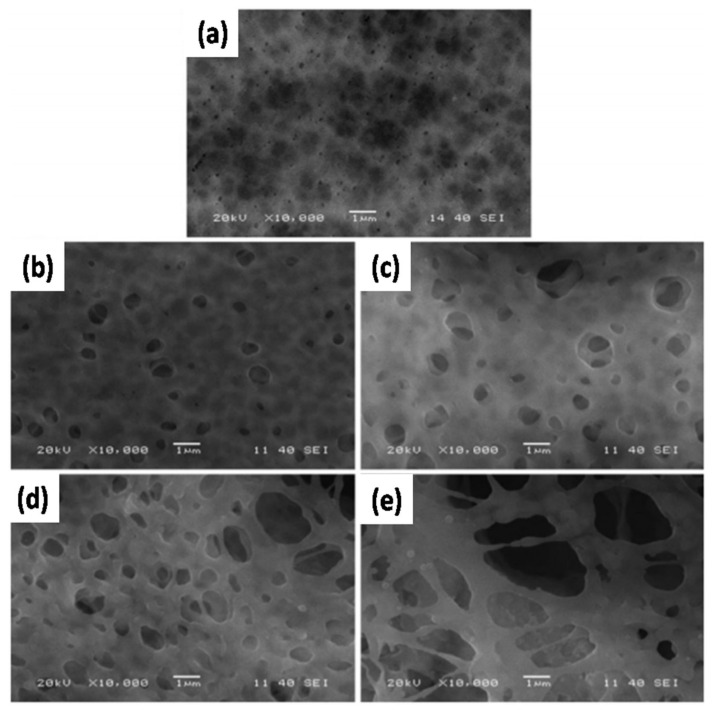
SEM images of the GPE membrane: (**a**) pristine PVDF, (**b**) PVDF-1% SiO_2_(Li^+^), (**c**) PVDF-2% SiO_2_(Li^+^), (**d**) PVDF-5% SiO_2_(Li^+^), and (**e**) PVDF-10% SiO_2_(Li^+^). Reprinted with permission from Li et al. [109]. Copyright 2013 Springer Nature.

**Figure 15:**
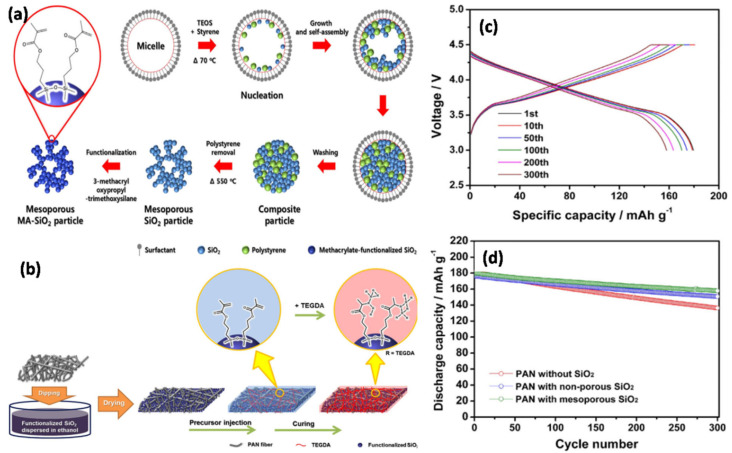
Reaction schemes for the synthesis of (**a**) mesoporous MA-SiO_2_ particles and (**b**) cross-linked composite GPE. (**c**) Charge-discharge curves of the cell and (**d**) cyclic performance with different electrolytes at 25 °C. Reprinted with permission from Kim et al. [111]. Copyright 2016 Springer Nature.

Li et al. [109] obtained similar results and proposed that the addition of SiO_2_(Li^+^) increases the amorphous phase and porosity of the polymer film, thus improving the adsorption and gelation of the LE. From the surface morphology of the membranes, as shown in Figure 14, it was established that as the content of SiO_2_(Li^+^) increases, the pore size of the film also increases, resulting in a high uptake of liquid electrolyte. The electrolytic conductance of the GPE was improved because of the large number of Li^+^ ions in SiO_2_(Li^+^). However, the GPE became very fragile when the SiO_2_(Li^+^) content reached 10 wt%. Manisankar et al. [110] synthesized superhydrophobic PVDF-SiO_2_ films with different SiO_2_ contents by electrospinning. When the SiO_2_ content increased, the surface roughness of the membrane also increased, but the average diameter of the nanofibers was not affected. Kim et al. [111] synthesized a secure and flexible electrolyte through the combination of mesoporous SiO_2_ NPs containing methacrylate groups and fibrous PAN membrane (Figure 15a,b). The initial discharge capacity delivered was 157.9 mAh g^−1^ after 300 cycles with a capacity retention of 88.0%. In addition, the GPE containing the mesoporous SiO_2_ particles exhibits better Li^+^ ion transfer than the GPE containing non-porous SiO_2_ particles (Figure 15c,d). This study emphasized the role of SiO_2_ mesoporous NPs compared with non-porous SiO_2_ NPs when attempting to achieve good electrochemical properties in terms of discharge capacity, capacity retention, rate capability, and cycling stability. GPEs containing a combination of PEO/LiClO_4_ complex and 1,3 dioxolane (DIOX)/tetraethyleneglycol dimethylether (TEGDME) as plasticizer with a SiO_2_ filler has also been synthesized [112]. An increase in SiO_2_ filler content and plasticizer reduced the degree of crystallization of the polymer membrane, thus increasing the ionic conductivity.

Wu et al. [22] studied the influence of SiO_2_ NP content on PVdF-HFP/IL membranes in terms of their ion conduction and discharge capacity in LIBs. The crystallization phase of the membrane was reduced due to the dispersion of SiO_2_ NPs, which hindered the structural stability of the polymer but improved ion transport because of their interaction with the amorphous phase of the host polymer. The SiO_2_ NPs acted as multifunctional inorganic fillers with good interfacial stability, which increased the ion conduction and Li^+^ ion transfer number for the poly(propylene carbonate)-based GPE in Li-S batteries [113].

Hu et al. [114] studied a GPE containing dispersed SiO_2_ NPs in a PEO matrix. Uniformly dispersed SiO_2_ NPs were obtained in the polymer matrix owing to the high miscibility of all the precursors. The synthesized GPE nanocomposite membrane significantly improved the electrochemical performance, which suggests a promising strategy for the development of safer and more flexible Li-metal batteries. The ionic conductivities and temperatures of the significant GPEs containing SiO_2_ fillers are listed in Table 3.

### 3.4. Zirconium Dioxide (ZrO_2_)

Vickraman et al. [115] studied a novel GPE containing lithium bis(oxalato)borate (LiBOB) as the Li salt and PVdF-PVC as the polymer matrix with varying contents of ZrO_2_. The high ionic mobility of the GPE is related to the large amorphous phase of the polymer host and its large free volumes that enhanced Li^+^ ion transfer. Suthanthiraraj et al. [116] investigated the effect of ZrO_2_ NPs in a new PPG-silver triflate (AgCF_3_SO_3_) system in terms of the improvement to ion transport and electrochemical behavior. This study showed that structural modification of the polymer matrix by the addition of ZrO_2_ NPs increased the ionic mobility and physicochemical properties of the GPE. Sivakumar et al. [117] reported the effect of the different concentrations of dispersed ZrO_2_ from the perspective of its enhanced ionic conductivity. The ionic conductivity increased upon the introduction of ZrO_2_ in the bare gel polymer system up to a loading of 6 wt%. However, a further increase in the ZrO_2_ content reduced the conductivity because of the larger crystalline region present in the matrix, which hindered the ionic mobility. Similar results were observed by Sivakumar et al. [118] demonstrating that the introduction of ZrO_2_ into a PVDF-HFP-(PC+DEC)-LiClO_4_ system significantly improved the electrolytic conductance of the GPE. The addition of ZrO_2_ NPs restricted the reorganization of the polymer chain structure, thereby increasing the amorphous phase of the polymer and improving the electrolytic conductance. Chen et al. [119] synthesized a novel GPE by in situ immobilization of ionic liquids (ILs) and nanoporous ZrO_2_ in a polymer matrix. This study showed that the ZrO_2_ skeleton cooperates with Li salts, resulting in improved dissociation of the Li salts and Li^+^ ion transfer. Therefore, a discharge capacity of 135.9 mAh g^−1^ was obtained after 200 cycles at 30 °C. In addition, the cell operated well in the temperature range of −10 to 90 °C. The good contact and stable interface between the Li-metal electrode and GPE can be attributed to its effective electrochemical performance in Li-metal batteries.

**Figure 16:**
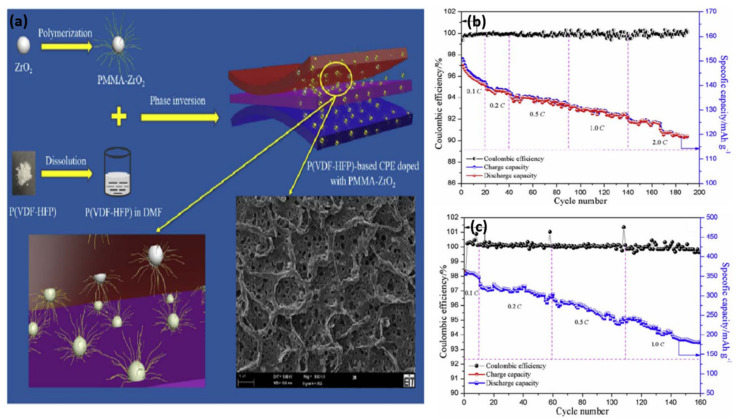
(**a**) Schematic of the GPE-sPZ hybrid particles. (**b**,**c**) Cyclic performance and Coulombic efficiency of the Li/CPE-sPZ/LiCoO_2_ and Li/CPE-sPZ/graphite coin cells at different C-rates at room temperature. Reprinted with permission from Xiao et al [120]. Copyright 2018 Elsevier B.V.

Xiao et al. [120] synthesized a novel GPE by combining synthesized PMMA-ZrO_2_ (sPZ) hybrid particles with a P(VDF-HFP) polymer matrix. The morphology of the GPE membrane containing homogeneously interconnected micropores is shown in Figure 16a. As shown in Figure 16b, the cell delivers 126.4 mAh g^−1^ at 2.0 C after 150 cycles, corresponding to a capacity retention of 85.2% at 0.1 C. The cell containing a graphite electrode delivers 288.5 mAh g^−1^ at 0.5 C after 80 cycles (Figure 16c). The modified ZrO_2_ significantly enhanced the properties of the GPE in terms of mechanical strength, ion conduction, and thermal stability.

Khoon et al. [121] obtained similar results and proposed that the ZrO_2_-based GPE has the potential to be applied in lithium polymer batteries owing to the improved Li^+^ ion transfer. The incorporation of ZrO_2_ NPs into the polymer-salt system resulted in a higher electrolytic conductance than that for the GPE without ZrO_2_ NPs. Prasanna et al. [122] prepared a nanocomposite GPE comprising a solution of zinc trifluoromethanesulfonate in a 1-ethyl-3-methylimidazolium bis(trifluoromethylsulfonyl)imide IL entrapped in a PVC/PEMA blend and dispersed ZrO_2_ nanofillers via a solution casting method. The GPE film showed the highest ionic conductivity of 3.63 × 10^−1^ mS cm^−1^ at room temperature when 3 wt% ZrO_2_ nanofiller was added. A list of significant GPEs containing ZrO_2_ fillers is provided in Table 4 with their ionic conductivities and temperatures.

### 3.5. Cerium Oxide (CeO_2_)

Rajendran et al. [123] reported a GPE system containing PEO-PMMA-LiClO_4_-DMP with varying CeO_2_ contents. The maximum ionic conductivity achieved was 2.07 × 10^−1^ mS cm^−1^ when 10 wt% CeO_2_ was added to the GPE. Vijayakumar et al. [124] prepared a new GPE membrane containing a PVDF-HFP-based polymer electrolyte and micro/nanosized CeO_2_ using a phase inversion technique. The highest ionic conductivity achieved was 2.47 × 10^0^ mS cm^−1^ at room temperature in the presence of 8 wt% CeO_2_. The addition of CeO_2_ NPs to the GPE reduced the interfacial resistance and enabled a wider electrochemical window and good cycling performance. Vijayakumar et al. [125] reported similar results in which the incorporation of CeO_2_ reduced the ion coupling and increased the charge carrier number, thereby improving the ionic conductivity. However, an excess of CeO_2_ led to an increased dilution effect, which resulted in a continuous decrease in the electrolytic conductance. Kumar et al. [126] designed a new class of nanocomposite polymer electrolytes to elucidate the origin and nature of the interactions between the surface of the CeO_2_ filler NPs in the polymer chain and the migrating ionic species. The study showed an enhanced percolation network among the CeO_2_ particles due to the increased ionic dynamics and surface interactions, which led to an increase in the electrolytic conductance. The surface interactions of the filler played an important role in increasing the charge carrier number and mobility, resulting in the highest ion conductivity of 5.2 × 10^−1^ mS cm^−1^ being achieved at room temperature. Polu et al. [127] synthesized a GPE based on PEG-Mg(CH_3_COO)_2_ as the polymer matrix and CeO_2_ NPs as the inorganic filler. The maximum ionic conductivity achieved was 3.40 × 10^−3^ mS cm^−1^ when 15 wt% CeO_2_ was added to the GPE system. This study indicated that the addition of a certain concentration of filler increased the ionic conductivity, but further addition resulted in a decrease in conductivity. A list of GPEs containing CeO_2_ fillers is presented in Table 5 along with their ionic conductivities and temperatures.

### 3.6. Barium Titanate (BaTiO_3_)

Kim et al. [128] studied the effect of BaTiO_3_ nanosized filler content in composite polymer electrolytes (CPEs). The optimum content of 15 wt% BaTiO_3_ showed reduced crystallinity of the CPEs and high ion conduction with a wide electrochemical window and good thermal stability. The results indicated that the addition of BaTiO_3_ filler affects the electrochemical properties and the crystallinity of the CPEs. Sivakumar et al. [129] synthesized a GPE by dispersing hydrothermally derived BaTiO_3_ NPs in PVC-PEMA-EC/DMC-LiClO_4_. The use of BaTiO_3_ NPs as fillers increased the electrolytic conductance owing to the improvement in the polymer fractions and the amorphous phase of the GPE. The presence of dispersed BaTiO_3_ NPs in the GPE prevented the growth of a passive layer on the surface of the Li-metal anode. Manimuthu et al. [130] discussed the influence of different ratios of BaTiO_3_ NPs on the PEO/PVDF-HFP-based polymer electrolyte. The presence of a BaTiO_3_ filler with high polarity reduced the crystallinity of the polymer because of the cooperation between the polymer chain and the filler surface, which increased the ion conduction of the GPE membrane. Table 6 lists BaTiO_3_ filler-based GPEs with their ionic conductivities and temperatures.

## 4. Mechanism of Li^+^ Transport on the Interface between the Inorganic Filler and Polymer Matrix in GPEs

As GPEs combine the advantages of LEs and SPEs in terms of electrolytic conductance and mechanical stability, they are considered to have more potential for practical applications in LIBs and non-LIBs [13]. During the charge–discharge process, a solid electrolyte interface (SEI) is still formed, similar to LEs, through a reaction between the plasticizers and electrode surface in GPEs. The electrochemical behavior and internal resistance allow the measurement of the ionic conductivity of the GPE at various charge–discharge rates [131]. Generally, LEs have high electrolytic conductance in the range of 10^−0^ to 10^1^ mS cm^−1^; thus, to evaluate the efficient usage of the GPE, its ionic conductivity should be higher than that of LEs (>10^−1^ mS cm^−1^) [42,132]. In gel-type polymer electrolytes, polymers are used as host matrices to trap the liquid constituent [133]. In this case, Li^+^ ion transfer is not affected by the segmental polymer chain motion but through the swollen gelled phase or liquid phase. In addition, the solvents used for GPEs should have a high dielectric constant and low viscosity. In SPE, polymer chains conduct local segmental motion continuously, leading to the formation of the free volume. Under the effect of the electric field, the lithium ions diffuse to a new coordinating position along the polymer chain or move from chain to chain through these free volumes. In the presence of ceramics, the diffusion of Li^+^ ion is enhanced because the ceramics increase the free volume of the polymer chain. Thus, the mechanism of reduction in the polymer crystallinity is not dependent on the chemical nature of the filler, but on the size, volume fraction, and shape of ceramic filler [134]. Because of the different particle sizes, the optimal content of ceramic fillers to achieve optimum diffusion of Li^+^ ion is different. Inert ceramic oxide fillers such as TiO_2_, SiO_2_ are often used with only a few percent (ceramic-in-polymer) to achieve optimum ionic conductivity. However, as the inert filler content increases, it decreases the mechanical strength of the polymer membrane. In contrast, active fillers, such as LLZO, LAGP, etc., have high ionic conductivity and can participate in Li^+^ ion transport, thus when the active filler content increases, the conductivity can be increased accordingly. Therefore, depending on the properties and content of the active filler and polymer, the optimal content to achieve maximum electrical conductivity is different [57]. The dissociation of the salt is facilitated by a high dielectric constant, while a low viscosity increases the ionic mobility in the electrolyte, resulting in a high electrolytic conductance [135]. In addition, the functional groups of the polymer matrix should be able to dissolve the Li salt to form the polymer-salt system. This requires that the lattice energy of the salt be relatively low, but the dielectric constant of the polymer matrix is relatively high. More importantly, the properties of the lithium salt will affect the performance of lithium polymer batteries. Because of its high ion conductivity, good electrochemical stability, and corrosion resistance for aluminum current collector, LiPF_6_ is mainly used in commercial LIBs. However, the halogen-based lithium salts have some disadvantages: LiClO_4_ is potentially explosive when exposed to an organic substance; LiAsF_6_ contains toxic As; LiBF_6_ and LiPF_4_ are easily decomposed into HF, which is toxic and corrosive in the moist atmosphere. Organic lithium salts, such as LiCF_3_SO_3_, LiTf, etc., are highly resistant to oxidation, thermally stable, and non-toxic, but they have poor ionic conductivity. LiTFSI is the best choice for Li salt in LIBs with high solubility in common solvents, but they are corrosive to aluminum current collectors. Therefore, the search for reliable new lithium salts has remained at the center of research in recent years [136]. Typically, electrolytic conductance is related to the elementary electric charge and ion mobility. The electrolytic conductance of GPEs is mainly dominated by the properties of the trapped liquid electrolyte in the micropores of the membrane; thus, the interconnected micropores of the membrane are the main factor affecting the mobile ions. In contrast, for a homogeneous membrane or a membrane without micropores, the Li^+^ ion mobility is primarily determined by the swollen gelled phase. In addition, an incomplete drying during the SPE preparation leads to the presence of moisture and solvent that results in the decomposition reaction of polymer. The standard drying process for the preparation of SPE membrane with a high content of Li salt (polymer-in-salt) will trap a large amount of solvent due to the strong interaction between solvent and ion species, which leads to the high ionic conductivity. The presence of trace water up to a few ppm remained after drying under vacuum was sufficient to trigger the depolymerization process. This process can have a strong impact on the electrochemical performance. Therefore, it is necessary to remove water to avoid the depolymerization of the polymer [137]. The possible mechanism of ion transport is as follows: (i) Li^+^ ions are located in the porous structure of the polymer; (ii) through dispersion of the inorganic filler, the porous structure of the polymer is maintained, which assists in the absorption of the LE in the GPE, resulting in alleviation of the leakage problem and an enhanced safety of the applied device; and (iii) Li^+^ ions migrate from one coordination site to a new site through the liquid phase or gelled phase (porous structure) via the effect of the inorganic fillers.

**Figure 17:**
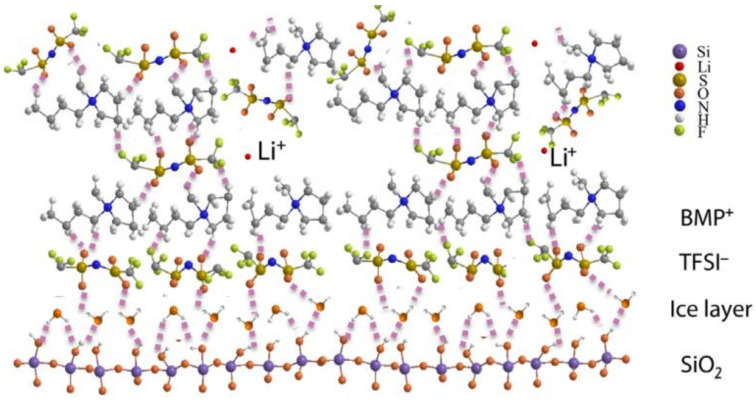
The model of the electrolytic conductance on the inorganic filler (SiO_2_) interfaces through the layers of immobile ice layer absorbed on OH-terminated silica surface. Reprinted with permission from Chen et al. [138]. 2020 Creative Commons Attribution-Noncommercial liscense.

A new concept has been recently proposed by Chen et al. [138] who explained the electrolytic conductance is highly affected by the layers of chemical water (immobile ice layer) adsorbed on OH-terminated SiO_2_ surface. They proposed the immobilization of 1-butyl-1-methylpyrrolidinium-bis TFSI (BMP TFSI) IL molecules extends the formation of ice layer on the SiO_2_ surface (Figure 17). In symmetric O=S=O groups on the TFSI anion, one oxygen can interact with a hydroxyl group on silica surface while other oxygen atom interacts with BMP cation groups. The TFSI anion in LiTFSI also has two groups of O=S=O, ensuring dense adsorption of the TFSI monolayer. However, the molecular interaction between the BMP cation and TFSI (LiTFSI) anion is different because TFSI has free rotation and no polarization from the underlying surface. The TFSI molecules must compensate for the positive bipolar charge due to the formation of H bonds between the O group of the TFSI anion and the OH group of the ice layer. This loosens the bond between the Li^+^ cation and the TFSI anion. In this way, the concentration of free Li^+^ increases at this interface, resulting in higher ionic conductivity.

**Figure 18:**
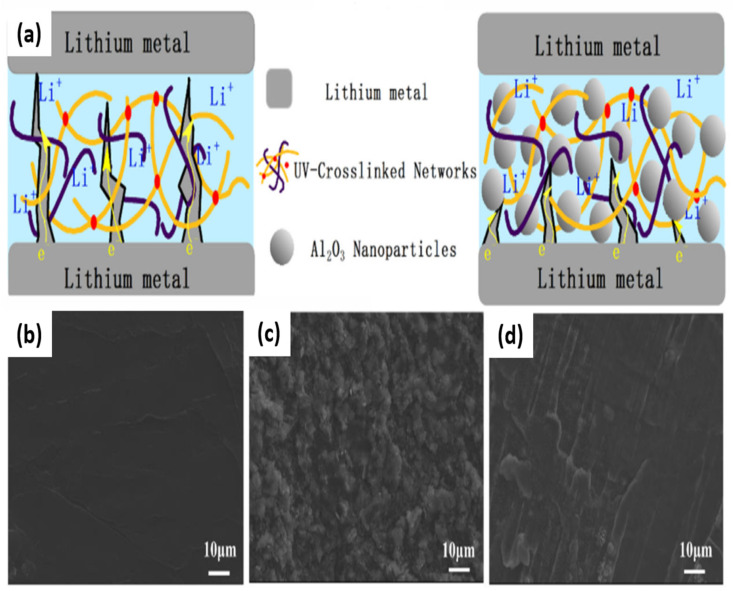
(**a**) Schematic illustrations the prevent of lithium dendrite growth by Al_2_O_3_-GPE electrolyte; (**b**) pristine of lithium anode before cycling in LFP/GPE/Li cell; (**c**) lithium anode in LFP/GPE/Li cell after 200 cycles at 0.5 C; (**d**) lithium anode in LFP/Al_2_O_3_-GPE/Li cell after 200 cycles at 0.5 C. Reprinted with permission from Liu et al. [143]. Copyright 2018. The Chinese Ceramic Society. Production and hosting by Elsevier B.V.

In addition to the Li^+^ ions transport mechanism, the interaction of lithium dendrite with the polymer and filler also needs to be considered. The dendrite growth during charge-discharge cycling hinders the utilization of GPE in LIBs, causing detrimental effects related to the safety and electrochemical performance of the battery, although its effect is lower than that of LE [139,140]. The inclusion of inorganic filler into GPE can suppress the formation of lithium dendrite through enhancement of the mechanical properties of the polymer matrix, trapping the LE resulting in a uniform Li^+^ ions flow at the Li metal-electrolyte interface [141]. Liu et al. studied the growth of Li dendrite through the addition of SiO_2_ NPs (nano-SiO_2_ and acid-modified nano-SiO_2_ filler) into PEO-LiTFSI system at current densities of 0.1 and 0.5 mA cm^−2^ [142]. The study showed that the interfacial resistance of cells is significantly reduced when introducing SiO_2_ NPs. However, more importantly, the acid-modified nano-SiO_2_ filler was the most effective to reduce the interfacial resistance. The acid modification on the nano-SiO_2_ surface immobilized the PEO chains and promoted the movement of Li^+^ ions. In addition, the Lewis acid–base interaction between the hydroxyl groups of the trace water in polymer electrolyte and the surface of acid-modified nano-SiO_2_ prevented the reaction of the traces water with Li metal, thereby hindering the growth of lithium dendrite. The interaction of Li dendrite with polymer and inorganic filler was also observed by Liu et al. [143]. In this study, they prepared a multi-functional GPE by combining ultraviolet (UV)-cured ethoxylated trimethylolpropane triacrylate (ETPTA) macromer with PEO and Al_2_O_3_ NPs as an inorganic filler. The uniform dispersion and high-density of Al_2_O_3_ NPs in GPE acted as a filler to stabilize the electrode interface as well as a protective agent to prevent the lithium dendrite growth, thus promoting good cycle stability (Figure 18a). From SEM images (Figure 18b–d), LiFePO_4_/GPE/Li cell showed large dendrites on the surface of lithium anode after 20 cycles at 0.5 C compared with the smooth surface of pristine lithium anode before cycling. In contrast, in the presence of Al_2_O_3_ NPs, no dendrites were observed on the surface of lithium anode in LiFePO_4_/Al_2_O_3_-GPE/Li cell. The addition of inorganic filler can suppress the lithium dendrite growth, which in turn greatly improves the safety of LIBs. Besides, the presence of polymer can prevent direct contact of the electrode and LE.

For SPE, the dendrite formation in the Li metal anode causes a short circuit of the battery, in which the polymer decomposition is the main failure mechanism of this battery [144]. Golozar et al. studied the mechanism of these dendrite formation [145]. The presence of carbon increases the hardness of the dendrite and facilitates their penetration into the SPE, thereby causing a short circuit of the battery. Li_x_C_x_ is formed through the reduction of SPE throughout the cycle, which produces carbon-rich species. In addition, the decomposition of LiTFSI at grain boundaries has also been observed [146]. The dissolution of the lithium metal into the polyether begins at the grain boundary and continues after many cycles leading to the depletion of lithium. Li_3_N initially formed becomes insoluble in the next cycles and further decomposition of salt leads to the formation of Li_2_S, LiC_x_F_y_, Li_x_CNF_3_, and Li_y_SO_x_, where LiF acts as a protective layer for the lithium from further dissolution.

## 5. Synthesis Methods for Inorganic Gel Polymer Electrolytes

### 5.1. GPEs Based on Physical Preparation Methods

GPE can be separated into two categories, physical and chemical gels, based on their preparation method. In physical gels, LEs are confined to the polymer matrix without significant polymer-solvent bonding. The GPE can be prepared from a dry polymer membrane that undergoes swelling by a liquid electrolyte containing Li salts and plasticizers. The general synthesis methods include conventional solution casting, phase inversion, and electrospinning.

In the solution cast method, the solvent should be able to dissolve both the Li salt and polymer matrix without water molecules absorbed from environment [42]. Forsyth et al. [147] demonstrated a purpose-designed Teflon mold method for the preparation of GPEs which combines an IL electrolyte (consisting of 3.8 m LIFSI in trimethyl(isobutyl)phosphonium bis(fluorosulfonyl)imide (P_111i4_FSI)), poly(diallyldimethylammonium) bis(trifluoromethanesulfony)imide (PDADMA TFSI), and Al_2_O_3_ NP inorganic filler. The P_111i4_FSI showed a wide electrochemical window, and the high concentration of Li salt could significantly enhance the lithium stripping and plating. On the other hand, the introduction of Al_2_O_3_ increased the mechanical stability, which allowed more IL to interact with the GPE. The optimized composition of GPE with 5 wt% Al_2_O_3_ NPs and 50% of IL showed the highest ionic conductivity of 0.28 × 10^0^ mS cm^−1^ and enhanced the Li^+^ ion transport. The solution cast method is a commonly used traditional method due to its ease of fabrication. The obtained GPEs generally show increased electrolytic conductance and good interfacial properties between electrode and electrolyte as well as the good electrochemical performance of the full cell polymer batteries.

Phase inversion method is also commonly used to fabricate highly porous polymeric film through a de-mixing procedure in which an initial homogeneous polymer solution is changed from a liquid state to a solid-state [148]. Liu et al. [149] integrated non-woven fabrics of PVDF-PAN-SiO_2_-based GPE membrane using a Loeb-Sourirajan (L-S) inverted phase method (i.e., the dry-wet phase inversion technique to prepare cellulose acetate membrane for seawater desalination) to obtain a polymer membrane with uniform pore size. The chemical reaction that occurred between two salts (NaHCO_3_ and CH_3_COOH) during the membrane preparation process promotes the formation of porous and interconnected structures in GPE. The GPE membrane showed the highest ionic conductivity of 3.32 × 10^0^ mS cm^−1^ with enhanced the mechanical strength and electrolyte uptake. The combination of L-S inverted phase and chemical reaction process was suitable to prepare polymer matrices in GPE. The facile electrolytic conductance and stable interfacial property between electrode and electrolyte in this GPE resulted in an excellent performance in LIBs.

Electrospinning is another common method to produce polymer fibers with a diameter ranging from tens of nanometers to tens of micrometers through the electrostatic repulsion of the polymer solution [150,151]. A novel PVDF-HFP-based GPE with nanostructured IL and SiO_2_ NP-tethered 1 methyl-1-propulpiperidinium bis(trifluoromethanesulfonyl) imide (SiO_2_PPTFSI) was prepared by electrospinning process [152]. The obtained GPE showed good mechanical stability, an increase in the electrolytic conductance as well as Li^+^ ion transfer. More importantly, the assembled cell showed an initial discharge capacity of 119 mAh g^−1^ and a capacity retention of 92.1% after 460 cycles at 1 C. The use of a nanostructured IL and modified SiO_2_ presented a potential candidate to improve the cyclic performance and the safety of LIBs. Although the electrospinning method is a cost-effective and simple manufacturing process, the difficulty in controlling the pore structure and the time-consuming process are significant drawbacks that need to be considered.

Generally, physical methods are used to cross-link polymer chains through weak physical interactions in GPEs. Owing to the weak interaction between the constituents, safety issues remain a concern as the polymer matrix can easily swell or dissolve in the LE at high temperatures, which leads to solvent leakage and reduced electrochemical performance. Poor thermal stability is another limitation when applying GPEs synthesized through physical methods to practical applications [131].

### 5.2. GPEs Based on Chemical Preparation Methods

The chemical methods used to prepare GPEs are also called “in situ synthesis” methods. In chemical gels, the crosslinking agent and functional groups of the polymers form a chemical bond. The precursor solution is prepared by dissolving the crosslinking agent and monomers in a LE in a specific ratio. Subsequently, the GPE is synthesized by polymerization of the monomer, forming a cross-linked network, and the LE is uniformly immobilized in the nanopores. Polyester is the most commonly used material for GPEs owing to the facile interaction between the lithium-ion and ethylene oxide (EO) units. Generally, polyether is formed when using precursors containing methacrylate groups on the surface [153]. Sato et al. [154] designed novel three-dimensional hybrid silica particles (PSiPs) with concentrated polymer brushes (CPB) with trace amount of IL. Initially, the surface of monodisperse silica particle (SiP) was changed by a mixture of ethanol/water/(2-bromo-2methyl) propionyloxyhexyltriethoxysilane (BHE) to form a new type of colloidal crystal. Then, by surface-initiated living radical polymerization (LRP) process, the PSiPs were successfully synthesized by grafting well-defined polymers on the surface to form the CPB-modified particles. The GPE with CPB-modified SiP provided an advanced ion conduction channel with an orderly and clearly defined structure. A newly designed GPE gave the chance for bipolar LIB device with a good electrochemical performance and made it possible to apply in practical use. Guo et al. [155] fabricated ionic liquid GPEs (ILGPEs) supported by active filler LAGP or inactive filler SiO_2_ for enhanced ionic conductivity and electrochemical performance. As for inorganic fillers, the optimized content of nano-SiO_2_ could decrease the crystalline phase of the host polymer as well as promote the Li-ion transport. Nevertheless, when the concentration of SiO_2_ exceeded the optimal value, the fillers impeded the effective migration of Li^+^ ion into the ILGPE. However, in the case of LAGP filler, it not only reduced the crystallinity but also improved the electrolytic conductance of the host polymer even when the concentration was greatly increased because LAGP is also the source of Li^+^ ion. The IL-GPE with 10 wt% LAGP showed high thermal stability and no flammability, which suggests it can be a promising electrolyte for the highly safe energy storage devices. Ma et al. [156] improved the ionic conductivity of GPE by LATP ceramic particles, which were dispersed in polymerized ionic liquids (PILs) as a polymer matrix, and LiTFSi as a source of Li^+^ ion. The PIL was synthesized using 1-vinyl-3-ethylimidazolium TFSI as a monomer and nonwoven polyethylene terephthalate (PET) as a polymer in GPE. The optimized content of LATP (10 wt%) exhibited good rate performance and capacity retention of 97% after 250 cycles at 60 °C, indicating that PIL-LiTFSI-LATP can yield superior cyclic performance at the high temperature. Wang et al. [157] incorporated two-dimensional (2D) silica NP into PIL to optimize the transport properties of GPE. The desired transport properties were due to the bonding of the grafted PIL and mesoporous structure of 2D silica nano filler with abundant, shorter, and continuous ion transport pathways. In addition, the 2D nanofiller could effectively control the ion transport trajectory through the surface contact orientations, thus leading to higher ion conductivity than the GPEs added with zero-dimensional (0D) or one-dimensional (1D) nanofiller. The assembled cell showed a discharge capacity of 135.8 mAh g^−1^ after 30 cycles at 60 °C, suggesting that their capacity and capacity retention are superior to cells using unmodified PIL/IL PE (50.0 mAh g^−1^). The ionotropic gelation method is a technique used to prepare micro-and nanoparticles, which are synthesized by adding anion polyelectrolyte solution with drop-by-drop manner to an acidic chitosan solution. Chitosan is ionotropically gelated to be proton conductive membranes, creating proton conductor sites in a single step. In contrast, the pre-formed chitosan in the solution is not highly conductive because of the limited Li^+^ diffusion through the polymer matrix [158,159]. Kim et al. prepared a multifunctional binder network by combining chitosan and reduced graphene oxide (rGO) to enhance the electrochemical performance of Li-sulfur batteries [160]. A homogeneous network formed by the reaction of chitosan with GO in an aqueous solution enhanced the redox system by trapping lithium polysulfides, reinforced the mechanical properties, and promoted ion/electron movement. This multifunctional network binder can be used for high-performance Li-S batteries. Zhao et al. used chitosan crosslinked with a carboxylic acid or acrylic acid molecules to form a compatible binder for both silicon and graphite [161]. The crosslinked chitosan lattice can effectively regulate the large volume change of silicon particles throughout the cycles. In this sense, chitosan network can be used as an effective binder in LIBs.

## 6. Promising Applications of GPEs in Various Battery Systems

The ionic conductivity enhancement and host polymer structure modification realized by the addition of inorganic fillers in GPE can be applied to improve the electrochemical performance of various battery systems.

### 6.1. Lithium-Ion Batteries

Because GPEs are highly resistant to electrochemical oxidation compared to LEs, GPEs are selected as potential electrolytes for practical applications in LIBs. Many studies have used inorganic fillers such as SiO_2_, TiO_2_, Al_2_O_3_, etc., to enhance the mechanical strength and improve the ionic conductivity of GPE in LIBs. Lewis acid–base interaction between a surface group of filler and ions appeared to be responsible for this role. Some new inorganic fillers that have been recently introduced in LIBs are aluminum oxyhydroxide (AlO(OH)_n_), graphene oxide (GO), clays, etc., which are expected to modify the structure of polymer matrix and thus enhance the Li^+^ ion transport. Stephen et al. [162] prepared a multifunctional GPE by combining a PVdF-HFP as a polymer matrix, lithium bis perfluorosulfonyl imide (LiN(CF_3_SO_2_)_2_) as a lithium salt, and AlO(OH)_n_ as an inorganic filler. The introduction of AlO(OH)_n_ not only increases the amorphous domain and acts as “solid plasticizer” to promote the Li^+^ ion transfer but also provides a good interfacial property towards Li-metal anode. The full cell with GPE membrane exhibited first discharge capacity of 127 mAh g^−1^ with a capacity retention of 98.4% after 20 cycles at 70 °C. Aravindan et al. [163] continued to employ AlO(OH)_n_ as an inert filler in PVdF-HFP and indicated that the introduction of 10% AlO(OH)_n_ in GPE membrane enhanced the ionic conductivity due to the interaction of Lewis acid–base between F atoms in PVdF-HFP and OH^−^ groups in filler. This interaction increased the amorphous domain by preventing reorganization of polymer chains, thus leading to the enhancement of the electrolyte uptake at ambient temperature condition. In addition, the study showed high cyclic stability with a capacity retention of 97.8% after 10 cycles. The advantages of using AlO(OH)_n_ over conventional inorganic fillers, such as TiO_2_, SiO_2_, Al_2_O_3_, etc., are mainly because of its ability to promote more dissociation of lithium salt, which leads to the enhanced number of charged carriers, ionic conductivity, and electrochemical performance. Chen et al. [164] prepared a high-performance battery using a PVDF-HFP-based GPE co-doped with PEO and GO via weak hydrogen bond interaction. The GPE showed a 3D porous network with superior ionic conductivity up to 2.1 × 10^0^ mS cm^−1^ and excellent cycling stability with a capacity retention of 92% after 2000 cycles at 5 C. The good electrochemical performance was due to the abundant oxygen-functional groups in GO sheets that interact with the copolymer (PVDF-HFP and PEO polymer) to form an amorphous phase and porous structure, which is beneficial for both Li^+^ and PF_6_^−^ intercalation/deintercalation kinetics. Zhao et al. [165] used GO as a filler to synthesize the homogeneous GPE for enhancing the electrochemical properties of the cell. The presence of GO enhanced the contact between the GPE and electrodes and formed a more stable SEI layer, leading to the enhancement of electrolytic conductance and the Li^+^ ion transfer of GPE. Thus, the discharge capacity and cyclic performance of battery were effectively improved. Liu et al. [166] designed a GPE by incorporating graphene fillers in the presence of PVDF as a host polymer, and LiPF_6_ as a source of Li^+^ ions. This study demonstrated the decrease in crystallinity of porous PVDF due to the homogeneously dispersed graphene in host polymer, resulting in the increased GPE electrolytic conductance from 1.85 × 10^0^ mS cm^−1^ in pure PVDF to 3.61 × 10^0^ mS cm^−1^ in the presence of 0.002 wt% graphene, and enhanced cyclic performance of the cell. Chen et al. [167] studied a GPE that consists of PAVM as a host polymer, LiPF_6_ as a source of Li^+^ ion, and GO quantum dots (GOQD) as an inorganic filler. The GOQD hinders the formation of ion-solvent clusters and immobilizes anions; as a result, the assembled LiFePO_4_/GPE/Li cell showed a good performance at high rates (up to 20 C) and exhibited capacity retention of 100% after 500 cycles. From these results, it can be expected that layered GO can significantly change the properties of the host polymer even with a very low content due to its highly oxidizing and hydrophilic nature. Compared with conventional ceramic nanofillers, GO has some advantages because of its tunable surface functionalities, high compatibility, and excellent dispersion with the polymer network. Dyartanti et al. [168] prepared a PVDF-PVP-based GPE containing an montmorillonite (MMT) nano-clay as a filler. The addition of MMT clay showed an increased porosity of host polymer and enhanced the uptake of LE, leading to an increased ionic conductivity of 5.61 × 10^0^ mS cm^−1^. The full cell showed good cyclic stability with a capacity retention of 97.7% after 48 cycles. The use of clay as an inorganic layered filler is beneficial because of its unique characteristics of the length scale (channel width = 16 Å), high cation exchange capacity, suitable interlayer charge, and a substantial specific surface area (~31.82 m^2^ g^−1^). Table 7 summarizes the electrochemical performance of LIBs with inorganic fillers added in GPEs.

### 6.2. Sodium-Ion Batteries

Because of their versatility, flexibility, and thermodynamic stability, SPEs have been selected as one of the most promising candidates for high safety sodium-ion batteries (SIBs). However, the low ion mobility in SPEs at room temperature hinders their practical application in SIBs. The addition of inorganic fillers to GPEs is an effective method to improve the ion conduction of electrolytes. Conventional fillers, such as SiO_2_, TiO_2_, Al_2_O_3_, and BaTiO_3_, etc., have been used to synthesize composite solid polymer electrolytes for sodium batteries [169,170,171]. Hwang et al. [172] fabricated a GPE containing a PEO-based polymer electrolyte, NaClO_4_, and nanosized TiO_2_ using a solution casting technique. The GPE, with a EO:Na ratio of 20:1 (w:w) and 5 wt% TiO_2_, showed the highest ionic conductivity of ~2.62 × 10^−1^ mS cm^−1^ at 60 °C and enhanced the stability of cyclic performance of the cell. This result can be explained by the positive effect of the TiO_2_ filler, which reduced the crystallinity of the polymer, thus enabling faster ionic transport. Zhang et al. [173] prepared a novel GPE based on PMMA, PEG, NaClO_4_, and α-Al_2_O_3_ which contained acidic surface sites. High electrolytic conductance (1.46 × 10^−1^ mS cm^−1^ at 70 °C), wide electrochemical stability window (4.5 V vs. Na^+^/Na), and good mechanical stability were achieved by the GPE. The reversible capacity reached 85 mAh g^−1^, corresponding to a capacity retention of 94.1%, even after 350 cycles, when coupled with a Na_3_V_2_(PO_4_)_3_ cathode. Kumar et al. [174] investigated a GPE containing SiO_2_ NPs dispersed in PVDF-HFP. This membrane was transparent, flexible, and free-standing, which makes it suitable for flexible SIBs. The material showed a high electrolytic conductance of 4.1 × 10^0^ mS cm^−1^ at ambient temperature and good thermal stability owing to the formation of space-charge layers between the SiO_2_ particles and the gel region. Liu et al. [175] synthesized a PVDF-HFP/PMMA-based GPE membrane containing a suitable number of β-Al_2_O_3_ NPs. The incorporation of PMMA into PVDF-HFP-based film improved the ionic conductivity due to the amorphous properties of PMMA which can promote the uptake of the LE and enhance the interaction of carbonyl-carbonate groups in MMA monomer and electrolyte, respectively. The GPE membrane showed a high electrolytic conductance of 2.39 × 10^0^ mS cm^−1^ and enhanced the electrochemical stability window up to 5.04 V. Besides, the full cell exhibited good electrochemical performance with the first discharge capacity of 94.1 mAh g^−1^ and capacity retention of 85% after 300 cycles at 0.5 C. Wang et al. [176] employed a sodium ion conductive Na_3_Zr_2_Si_2_PO_12_ (NZSPO) in modified PVDF-HFP/PMMA/polyurethane (TPU)-based GPE to enhance the properties of membrane. The introduction of filler increased the amorphous phase and boosted the porosity of GPE membranes, leading to the increase in LE uptake. Besides, the NZSPO itself was the active filler; thus, it could provide pathways of ions at the interface between the filler and GPE. The GPE film showed ionic conductivity of 2.83 × 10^0^ mS cm^−1^ and a wide electrochemical window of 5.16 V. The full cell exhibited the first discharge capacity of 92.7 mAh g^−1^ with capacity retention of 99.2% after 100 cycles at 0.5 C. Yi et al. [177] prepared a PMMA-based GPE by introducing Na_3_Zr_2_Si_2_PO_12_ and PVDF-HFP to boost the interfacial adhesion between electrode and electrolyte. The GPE membrane showed a high electrolytic conductance of 2.78 × 10^0^ mS cm^−1^ and a wide electrochemical window of 4.9 V. More importantly, the assembled full cell with GPE exhibited the first discharge capacity of 96 mAh g^−1^ with excellent cyclability during 600 cycles.

Recently, inorganic NPs have also been employed to further enhance the electrochemical properties of PEO/Na-salts/ILs in GPEs. Song et al. [178] developed a hybrid GPE consisting of PEO-NaClO_4_-SiO_2_ and 1-ethyl-3-methylimidazolium bis(fuorosulfonyl)imide (Emim FSI) for sodium batteries. This GPE demonstrated an integrated structure by redox processes and interactions among the Emim FSI, silicon, and PEO. The GPE showed a high electrolytic conductance of 1.3 × 10^0^ mS cm^−1^ at ambient temperature and stable voltage window of 4.2 V vs. Na/Na^+^, which is sufficient for most cathode materials in SIBs. These results indicated that the introduction of nanosized inorganic fillers is a good strategy to enhance the electrochemical performance of polymer electrolytes. Table 8 presents the electrochemical performance of SIBs with GPEs containing inorganic fillers.

### 6.3. Magnesium-Ion Batteries

Magnesium (Mg) is the eighth most abundant element in the Earth’s crust, the third most abundant element in seawater, and is geographically widespread [179]. Compared with Li-metal anodes, it is less likely that Mg dendrites would grow as they thermodynamically prefer three dimensional crystal growth rather than one-dimensional growth [180]. Nevertheless, the presence of dendrite Mg has been reported in several LEs and remains the hurdle to overcome with Mg metal anodes. Mg metal interacts more strongly with counter ions or polymer matrices than lithium metal and requires a higher under/overpotential for Mg electrode position/dissolution. Therefore, it is necessary to develop new Mg polymer electrolytes [181]. Kim et al. [182] prepared Mg^2+^ ion-conducting polymer electrolytes containing P(VdF-co-HFP), Mg(ClO_4_)_2_-EC/PC, and SiO_2_ filler. This GPE achieved an electrolytic conductance of 3.2 × 10^0^ mS cm^−1^ at room temperature. Hashmi et al. [183] investigated a novel GPE nanocomposite based on PVdF-HFP containing dispersed MgO NPs. The maximum electrolytic conductance was 8 × 10^0^ mS cm^−1^ at room temperature when 3 wt% MgO was introduced. The assembled V_2_O_5_/GPE/Mg battery showed the low first discharge capacity of 58 mAh g^−1^ and poor cycling performance with capacity retention of 38% after 10 cycles, which are attributed to high interfacial resistance between Mg and GPE. Pandey et al. [184] studied the effect of MgO and SiO_2_ particle sizes in a PVDF-HFP-based polymer electrolyte. High conductivities of 1 × 10^1^ mS cm^−1^ for 3 wt% and 9 × 10^0^ mS cm^−1^ for 15 wt% SiO_2_ were obtained for the SiO_2_ dispersed gel electrolyte. The presence of MgO formed space-charge regions that facilitated the Mg^2+^ ion motion, thereby enhancing the electrolytic conductance. Hashmi et al. [185] investigated the effect of SiO_2_ NPs in a PVDF-HFP-based polymer electrolyte. The highest electrolytic conductance achieved was 1.1 × 10^1^ mS cm^−1^ at 25 °C when 3 wt% SiO_2_ NPs were added. The assembled full cell with GPE exhibited a first discharge capacity of 175 mAh g^−1^ with poor cyclability after 10 cycles. They also studied the effect of microsized MgO particle dispersion in a PVDF-HFP-based magnesium-ion (Mg^2+^) conducting GPE [186]. The maximum ionic conductivity reached 6 × 10^0^ mS cm^−1^ at room temperature with the incorporation of 10 wt% MgO particles. Hashmi et al. [187] reported a novel GPE membrane containing PVDF-HFP as the polymer matrix, Mg trifluoromethanesulfonate (Mg-triflate or Mg(Tf)_2_) in a mixture of EC and PC as the Mg salt and nanosized passive Al_2_O_3_ filler or active filler Mg aluminate (MgAl_2_O_4_). The presence of the filler increased the porosity of the membrane, thereby increasing the electrolytic conductance of the GPE film. The highest ionic conductivities achieved were 3.3 × 10^0^ and 4.0 × 10^0^ mS cm^−1^ when adding 30 wt% Al_2_O_3_ and 20 wt% MgAl_2_O_4_ fillers, respectively. To achieve the impressive improvement of electrochemical performance with the inorganic fillers-added GPE, it is thought that high impedance at the interface between Mg metal and GPE needs to be resolved, which requires the finding of suitable electrode materials. Table 9 shows the list of electrochemical performance of MIBs with inorganic filler-added GPEs.

### 6.4. Zinc-Ion Batteries

Because of the favorable features of Zn metal, such as low cost, low toxicity, and high natural abundance, Zn has received intensive attention as an anode material in Zn rechargeable batteries. Hashmi et al. [188] prepared a GPE containing PVDF-HFP as the host polymer, a solution of EC-PC-Zn(Tf)_2_, and nanosized ZnO filler particles. The GPE exhibited good thermal stability and good electrochemical performance achieving an electrolytic conductance of >10^0^ mS cm^−1^. LE immobilization and dispersion of the ZnO NPs in the GPE led to appropriate changes in the PVDF-HFP and filler-polymer interactions. Suthanthirarai et al. [189] prepared new GPE using a solution casting technique and evaluated the transport mechanism of a Zn^2+^-conducting polymer electrolyte system. The highest electrolytic conductance was 3.4 × 10^−3^ mS cm^−1^ at room temperature when 5 wt% TiO_2_ NPs were added. The presence of TiO_2_ NPs formed a space-charged region, which significantly enhanced the movement of the Zn^2+^ ions, thereby increasing the ionic conductivity. From good thermal stability and enhanced ionic conductivity achieved by adding inorganic fillers in GPEs, the choice of appropriate inorganic filler in GPE can be seen as a potential approach for the high-performance ZIBs.

## 7. Conclusions and Perspectives

The incorporation of inorganic fillers into the polymer/salt system has been demonstrated as a promising strategy to enhance the electrochemical performance of GPEs in the last few decades. The introduction of inorganic fillers improves the electrolytic conductance as well as mechanical and thermal stability of gel-state polymer electrolytes. In this review, a historical overview of the developments in GPEs is first provided and subsequently detailed fillers applied in GPEs are discussed. The possible mechanisms behind the conductivity enhancement of inorganic fillers are also briefly discussed. Finally, inorganic filler/polymer GPEs studied for use in various battery systems, including Li-, Na-, Mg-, and Zn-ion batteries, were reviewed.

Although there have been several studies regarding the mechanisms behind the ionic conductivity enhancement and improvement in electrochemical stability with the addition of fillers, further fundamental understanding should be continuously pursued with novel composite polymer electrolyte designs for the successful implementation of GPEs in high-performance lithium batteries, especially for industrial applications. To achieve this, both experimental and theoretical calculation approaches should be synergistically combined. On the experimental side, it is important to find the optimal composite GPE structure, wherein the interfacial volume between the polymer and filler is maximized and agglomeration of the constituents is minimized. This structural feature will be beneficial for ionic conductivity and electrochemical stability, thus improving the cyclic stability in various battery applications. Consideration of the appropriate interfacial structure between the gel electrolyte and the electrode is another important factor. Long and tortuous ion pathways greatly inhibit the utilization of active materials in the performance of Li-polymer batteries. A reduced internal and interfacial resistance at the cathode and electrolyte/electrode interface can significantly enhance the cycling performance and rate capability of LIBs, resulting in a higher energy density. Besides, optimization of the active material loading (mg·cm^−2^) is another important consideration to realize the high energy density of the cell. Although a high thickness film which accompanies the high mass loading can increase the energy storage capacity of LIBs, it does not necessarily result in the high energy density. Since the energy density is proportional to the specific capacity, it is necessary to maximize the specific capacity to obtain the high energy density. Normally, when the film thickness is excessively high, the specific capacitance decreases due to the inefficient electrolyte ion diffusion through the electrode film. If the film thickness is too low, the reproducibility of measured capacity becomes degraded. Therefore, the appropriate film thickness (or mass loading) is required to increase the energy density. In previous studies with GPEs, the best mass loading condition is in the range of 0.35 to 3 mg cm^−2^ [104,114,120,155,156,157,165,175,176]. In the theoretical calculations, a precise prediction of the overall structure of the constituents in the composite gel will be advantages in the design of GPE components; in particular, it can provide useful information regarding the appropriate content of inorganic filler for a given GPE system.

Finally, the preparation method utilized for GPEs needs to be considered in terms of simplicity and manufacturing costs. To apply newly developed GPEs to batteries in industry, facile and low-cost process should be utilized to prepare the GPEs. In addition, advanced fabrication approaches should be developed to apply the new GPE to micro-batteries and flexible devices.

## Figures and Tables

**Table 1:** List of GPEs with TiO_2_ filler with their conductivity and temperature.

Polymer	Salt/Plasticizers	Solvent	Conductivity (mS cm^−1^)	Temperature (°C)	Reference
PEO	LiClO_4_	ACN	-	90	[73]
PEO	LiBF_4_	ACN	7 × 10^−4^	30	[67]
PVDF-HFP	LiPF_6_-EC/DMC	Acetone	10^0^	25	[74]
PEG	LiClO_4_	Dicloro-methane	-	120	[75]
P(VdF-HFP)/P(EO-EC)	LiCF_3_SO_3_	Acetone	4.7 × 10^0^	25	[76]
PEO	LiClO_4_	THF	1.03 × 10^−2^	30	[77]
P(VdF-HFP)/P(EO-EC)	LiCF_3_SO_3_	Acetone	5.1 × 10^−2^	25	[78]
PAN/PEGDA	LiPF_6_/LiCF_3_SO_3_-EC/DMC	-	3.8 × 10^0^	30	[79]
PVDF-HFP	LiPF_6_-EC/DMC	Acetone + DBP	8.5 × 10^−1^	25	[80]
PAN	LiPF_6_-TMS	DMF	9.8 × 10^−1^	25	[81]
Cellulose acetate (CA)	NH_4_BF_4_	DMF	1.37 × 10^1^	30	[83]
PVDF/PMMA	LiClO_4_-EC/PC	DMF + acetone	3.9 × 10^0^	30	[84]
PEO-PVC	LiClO_4_	Cyclohexanone	8.33 × 10^−4^	-	[86]
PVA/PEGME	LiCF_3_SO_3_	DMSO	1.58 × 10^−1^	30	[87]
PVDF-HFP/PMMA	LiPF_6_-EC/DMC	DMF + Acetone	2.49 × 10^0^	30	[88]
Polyester diacrylate	LiClO_4_-EC	Benzoyl peroxide	1.8 × 10^0^	20	[89]
PAN/PVA	LiPF_6_-EC/DMC/DEC	Dimethylacet-amide	4.5 × 10^0^	30	[90]
PVDF-HFP	LiClO_4_-EC	Acetone	1.11 × 10^1^	30	[91]
PVC/PEMA	LiClO_4_-EC/DMC	THF	0.5 × 10^1^	30	[92]
PEMA	NaI-EC	THF	2.42 × 10^−1^	30	[93]

**Table 2:** List of GPEs with Al_2_O_3_ filler with their conductivity and temperature.

Polymer	Salt/Plasticizers	Solvent	Conductivity (mS cm^−1^)	Temperature (°C)	Reference
PVDF-HFP	LiPF_6_-EC/DMC DBP	NMP	1.95 × 10^−0^	25	[94]
PVDF-HFP	LiPF_6_-PC/DBP	Acetone	-	20–70	[95]
PEO/PMA	LiTFSI-EC/EMITFSI	-	10^−1^	25	[96]
PVA	NH_4_SCN	DMSO	5.81 × 10^1^	30	[97]
P(MMA-AN-EA)	LiPF_6_-EC/DMC	DMF	2.2 × 10^0^	30	[98]
PVFM	LiPF_6_-EC/DMC	NMP	4.13 × 10^−1^	25	[99]
PVDF-HFP	LiPF_6_-EC/DMC	Acetone-NMP	4.1 × 10^0^	25	[100]
PVDF	LiPF_6_-EC/PC	NMP	-	-	[101]
PEO	CF_3_COONa	ACN	-	-	
PAN	NaF-EC	DMF	4.82 × 10^0^	30	[103]
PVDF-HFP	NaClO_4_-EC/DEC	Acetone + ethanol	7.13 × 10^−1^	25	[104]
PVdF-HFP/PMMA	NaCF_3_SO_3_-EC/PC	THF + Acetone	1.5 × 10^0^	25	[105]

**Table 3:** List of GPEs with SiO_2_ filler with their conductivity and temperature.

Polymer	Salt/Plasticizers	Solvent	Conductivity (mS cm^−1^)	Temperature (°C)	Reference
PEO	LiClO_4_	DMF	3 × 10^−3^	30	[106]
PMMA	LiClO_4_-PC	DMF	5.64 × 10^−2^	80	[107]
PVdF-HFP	LiPF_6_-EC/DEC/DBP	Acetone	-	-	[108]
PVDF	LiPF_6_-EC/DEC/DBP	DMF	3.87 × 10^1^	30	[109]
PVDF	LiPF6-EC/DMC	DMF	7.73 × 10^−4^	30	[110]
PAN	-	DMF	1.8 × 10^−3^	25	[111]
PEO	LiCLO_4_-DIOX/TGDME	ACN	10^−4^	25	[112]
PVdF-HFP	LiTFSI-SDS	-	1.22 × 10^−3^	25	[22]
PPC	LiTFSI + LiNO_3_	DMAC	1.64 × 10^−4^	23	[113]
PEO	LiClO_4_	ACN	1.1 × 10^−4^	30	[114]

**Table 4:** List of GPEs with ZrO_2_ filler with their conductivity and temperature.

Polymer	Salt/Plasticizers	Solvent	Conductivity (mS cm^−1^)	Temperature (°C)	Reference
PVDF/PVC	LiBOB-EC/DEC	THF	1.53 × 10^0^	70	[115]
PPG	AgCF_3_SO_3_	THF	2.9 × 10^0^	30	[116]
PVdF-HFP	LiTFSI-PC	THF	4.46 × 10^0^	30	[117]
PS/PMMA	LiClO_4_	-	2.2 × 10^0^	30	[118]
PVDF-HFP	LiPF_6_-EC/DMC/EMC	DMF	3.6 × 10^0^	30	[120]
PVDF-HFP/MG49	LiBF_4_	THF	3.39 × 10^0^	30	[121]
PVC/PEMA	Zn(OTf)_2_	DMF	3.63 × 10^−1^	30	[122]

**Table 5:** List of GPEs with CeO_2_ filler with their conductivity and temperature.

Polymer	Salt/Plasticizers	Solvent	Conductivity (mS cm^−1^)	Temperature (°C)	Reference
PMMA/PEO	LiClO_4_-DMP	THF	20.65 × 10^−2^	30	[123]
PVDF-HFP	LiClO_4_-EC/DMC	NMP	2.47 × 10^0^	30	[124]
PVDF-HFP	LiClO_4_-EC/DMC	NMP	3.84 × 10^0^	30	[125]
PPG	AgCF_3_SO_3_	-	5.2 × 10^−1^	30	[126]
PEG	Mg(CH_3_COO)_2_	Distilled water	3.4 × 10^−3^	25	[127]

**Table 6:** List of GPEs with BaTiO_3_ filler with their conductivity and temperature.

Polymer	Salt/Plasticizers	Solvent	Conductivity (mS cm^−1^)	Temperature (°C)	Reference
PEO-PVDF	LiClO_4_-PC	ACN	1.2 × 10^−1^	30	[128]
PVC-PEMA	LiClO_4_-EC/DMC	THF	0.61 × 10^1^	30	[129]
PEO/PVDF-HFP	LiClO_4_-PC	Acetone	6 × 10^0^	30	[130]

**Table 7:** Lists of GPEs and their electrochemical performance in lithium-ion batteries (LIBs).

Polymer	Salt/Plasticizers/Fillers	Specific Capacity (mA h g^−1^)	Capacity Retention (%)	Long-Term Cycling	Current Density	Reference
PAN/PEGDA	LiPF_6_ -LiCF_3_SO_3_/EC-DMC/TiO_2_	138.0	-	50	0.2 C	[79]
PAN	LiPF_6/_TMS/TiO_2_	345.0	-	20	0.2 C	[81]
PAN	LiPF_6/_TMS/TiO_2_-SiO_2_	182.0	75.8	100	0.2 C	[82]
PVDF-HFP	LiPF_6_/TiO_2_	122.0	92.4	100	0.5 C	[85]
PVDF-HFP/PMMA	LiPF_6/_EC-DMC/TiO_2_	173.2	92.1	50	0.2 C	[88]
PAN/PVA	LiPF_6/_EC-DMC-DEC/TiO_2_	60.0	71.0	1000	20 C	[90]
P(MMA-AN-EA)	LiPF_6/_EC-DMC/Al_2_O_3_	132.0	94.8	100	0.2 C	[98]
PVFM	LiPF_6/_EC-DMC/Al_2_O_3_	140.3	88.6	15	0.2 C	[99]
PVDF-HFP	LiPF_6/_EC-DMC/Al_2_O_3_	160.2	96.0	50	0.1 C	[100]
PVDF-HFP	LiPF_6/_EC-DEC-DBP/SiO_2_	149.0	97.4	100	0.5 C	[108]
PAN	SiO_2_	157.9	88.0	300	0.5 C	[111]
PPC	LiTFSI-LiNO_3_/SiO_2_	597.0	85.0	500	0.1 C	[113]
PEO	LiClO_4_/SiO_2_	123.5	70.0	90	0.2 C	[114]
PVDF-HFP	LiPF_6/_EC-DMC-EMC/ZrO_2_	126.4	85.2	150	2 C	[120]
PVDF-HFP	LiClO_4/_EC-DMC/CeO_2_	105.8	86.0	30	0.5 C	[124]
PVDF-HFP	LiClO_4/_EC-DMC/CeO_2_	116.4	81.6	50	0.5 C	[125]
PEO/PVDF-HFP	LiClO_4/_PC/BaTiO_3_	123.0	-	100	0.3 C	[130]
PVDF-HFP	LiN(CF_3_SO_2_)_2_/AlO(OH)_n_	125.0	98.4	20	0.1 C	[162]
PVDF-HFP	LiBOB/AlO(OH)_n_	157.0	97.8	10	0.1 C	[163]
P(VDF-HFP)-co-PEO	LiPF_6_/GO	103.0	92.0	2000	5 C	[164]
PVDF-HFP	LiTFSI/GO	120.0	94.4	100	0.2 C	[165]
PVDF	LiPF_6_/Graphene	144.0	96.6	100	2.0 C	[166]
PAVM	LiPF_6_/GOQD	-	100	500	5 C	[167]
P(OPal-MMA)	LiClO_4_/Clay	146.4	-	50	0.5 C	[168]

**Table 8:** Lists of GPEs and their electrochemical performance in SIBs.

Polymer	Salt/Plasticizers/Fillers	Specific Capacity (mA h g^−1^)	Capacity Retention (%)	Long-Term Cycling	Current Density	Reference
PVDF-HFP	NaClO_4/_EC-DEC/Al_2_O_3_	120.0	95.3	1000	1 C	[104]
PVDF-HFP/PMMA	NaCF_3_SO_3/_EC-PC/Al_2_O_3_	360.0	90.0	10	-	[105]
PEO	NaClO_4_/TiO_2_	45.0	91.5	25	0.1 C	[172]
PMMA-PEG	NaClO_4_/Al_2_O_3_	85.0	94.1	350	0.5 C	[173]
PVDF-HFP	NaCF_3_SO_3_/SiO_2_	21.0	15.0	8	-	[174]
PVDF-HFP/PMMA	NaClO_4_/β-Al_2_O_3_	80.0	85.0	300	0.5 C	[175]
PVDF-HFP/PMMA/TPU	NaClO_4_/NZSPO	92.0	99.2	100	0.5 C	[176]
PVDF-HFP/PMMA	NaPF_6_/NZSPO	96.0	-	600	1 C	[177]
PEO	NaClO_4_/SiO_2_	46.2	51.0	100	0.5 C	[178]

**Table 9:** Lists of GPEs and their electrochemical performance in MIBs.

Polymer	Salt/Plasticizers/Fillers	Specific Capacity (mA h g^−1^)	Capacity Retention (%)	Long-Term Cycling	Current Density	Reference
PVDF-HFP	Mg(ClO_4_)_2_/EC-PC/SiO_2_	24.0	41.4	11	-	[182]
PVDF-HFP	Mg(ClO_4_)_2_/EC-PC/SiO_2_	175.0	79.5	10	0.1 C	[185]
PVDF-HFP	Mg(ClO_4_)_2_/EC-PC/MgO	175.0	67.31	10	-	[186]
